# Retinal Molecular Changes Are Associated with Neuroinflammation and Loss of RGCs in an Experimental Model of Glaucoma

**DOI:** 10.3390/ijms22042066

**Published:** 2021-02-19

**Authors:** José A. Fernández-Albarral, Juan J. Salazar, Rosa de Hoz, Eva M. Marco, Beatriz Martín-Sánchez, Elena Flores-Salguero, Elena Salobrar-García, Inés López-Cuenca, Vicente Barrios-Sabador, Marcelino Avilés-Trigueros, Francisco J. Valiente-Soriano, Juan A. Miralles de Imperial-Ollero, Manuel Vidal-Sanz, Alberto Triviño, José M. Ramírez, Meritxell López-Gallardo, Ana I. Ramírez

**Affiliations:** 1 Instituto de Investigaciones Oftalmológicas Ramón Castroviejo, Grupo UCM 920105, Universidad Complutense de Madrid, 28040 Madrid, Spain; joseaf08@ucm.es (J.A.F.-A.); jjsalazar@med.ucm.es (J.J.S.); rdehoz@med.ucm.es (R.d.H.); elenasalobrar@med.ucm.es (E.S.-G.); inelopez@ucm.es (I.L.-C.); atrivino@med.ucm.es (A.T.); ramirezs@med.ucm.es (J.M.R.); 2 Departamento de Inmunología, Facultad de Óptica y Optometría, Oftalmología y ORL, Universidad Complutense de Madrid, 28037 Madrid, Spain; 3 Departamento de Genética, Facultad de CC, Biológicas, Fisiología y Microbiología, Grupo UCM 951579, Universidad Complutense de Madrid, 28040 Madrid, Spain; emmarco@bio.ucm.es; 4 Departamento de Fisiología, Facultad de Medicina, Grupo UCM 951579, Universidad Complutense de Madrid, 28040 Madrid, Spain; beatrm14@ucm.es (B.M.-S.); elenaflo@ucm.es (E.F.-S.); 5 Servicio de Endocrinología, Hospital Infantil Universitario Niño Jesús, Centro de Investigación Biomédica en Red de Fisiopatología, Obesidad y Nutrición, 28009 Madrid, Spain; vicente.barriossa@salud.madrid.org; 6 Departamento de Oftalmología, Facultad de Medicina, Instituto Murciano de Investigación Biosanitaria Virgen de la Arrixaca, Universidad de Murcia, 30120 Murcia, Spain; marcelin@um.es (M.A.-T.); fjvaliente@um.es (F.J.V.-S.); juanantonio.miralles@um.es (J.A.M.d.I.-O.); manuel.vidal@um.es (M.V.-S.); 7 Departamento de Inmunología, Facultad de Medicina, Oftalmología y ORL, Universidad Complutense de Madrid, 28040 Madrid, Spain

**Keywords:** cytokines, BDNF, VEGF, fractalkine, glaucoma, ocular hypertension, microglia, retinal ganglion cells (RGCs), Iba-1, Brn3a

## Abstract

Signaling mediated by cytokines and chemokines is involved in glaucoma-associated neuroinflammation and in the damage of retinal ganglion cells (RGCs). Using multiplexed immunoassay and immunohistochemical techniques in a glaucoma mouse model at different time points after ocular hypertension (OHT), we analyzed (i) the expression of pro-inflammatory cytokines, anti-inflammatory cytokines, BDNF, VEGF, and fractalkine; and (ii) the number of Brn3a+ RGCs. In OHT eyes, there was an upregulation of (i) IFN-γ at days 3, 5, and 15; (ii) IL-4 at days 1, 3, 5, and 7 and IL-10 at days 3 and 5 (coinciding with downregulation of IL1-β at days 1, 5, and 7); (iii) IL-6 at days 1, 3, and 5; (iv) fractalkine and VEGF at day 1; and (v) BDNF at days 1, 3, 7, and 15. In contralateral eyes, there were (i) an upregulation of IL-1β at days 1 and 3 and a downregulation at day 7, coinciding with the downregulation of IL4 at days 3 and 5 and the upregulation at day 7; (ii) an upregulation of IL-6 at days 1, 5, and 7 and a downregulation at 15 days; (iii) an upregulation of IL-10 at days 3 and 7; and (iv) an upregulation of IL-17 at day 15. In OHT eyes, there was a reduction in the Brn3a+ RGCs number at days 3, 5, 7, and 15. OHT changes cytokine levels in both OHT and contralateral eyes at different time points after OHT induction, confirming the immune system involvement in glaucomatous neurodegeneration.

## 1. Introduction

Glaucoma is a multifactorial neurodegenerative disease, characterized by progressive damage of the optic nerve produced by retinal ganglion cells’ (RGCs) death [1]. One of the most important treatable risk factors is the increase in intraocular pressure (IOP). However, IOP control cannot always prevent RGCs’ death [2]; thus, other mechanisms such as neuroinflammation may be involved in the glaucomatous damage progression [3,4]. The microglial cells are the immunocompetent cells of the central nervous system and respond to neuronal damage through activation [5]. In the course of this activation, microglial cells change their morphology (increase the cell body size, retract and reorient their processes, and can be transformed into amoeboid cells, acting as macrophages), proliferate, and migrate towards damaged areas [6,7,8,9]. In addition, activated microglia can alter the expression of different receptors such as cell surface transmembrane glycoprotein CD200 receptor (CD200R), receptor for the CX3C chemokine fractalkine (CX3CR1), and purinergic receptor P2Y12 (P2RY12) and can modify their gene expression, promoting the synthesis and release of inflammatory cytokines (tumor necrosis factor alpha (TNF-α), interleukin 1 beta (IL-1β), interleukin 6 (IL-6), etc.) [10,11,12]. These cytokines may be involved in the apoptosis of RGCs activated by both intrinsic and extrinsic pathways, exacerbating the neurodegenerative process [13,14]. In the experimental models of glaucoma and in the aqueous humor, serum, and retina of glaucoma patients, an increase in inflammatory mediators (IL-1β, interleukin 4 (IL-4), IL-6, interleukin 10 (IL-10), interleukin 12 (IL -12), interferon gamma (IFN-γ), vascular endothelial growth factor (VEGF), and TNF-α) has been found [15,16,17,18].

Activated microglial cells, such as macrophages, show two M1 and M2 activation phenotypes. M1 produces an intense inflammatory response and is characterized by the release of inflammatory mediators (nitric oxide and reactive oxygen species) and pro-inflammatory cytokines (TNF-α, IL-1β, IL-6, and IL-12). Uncontrolled activation of the M1 phenotype can induce chronic inflammation, which leads to neuronal death. However, the M2 phenotype is characterized by the release of neurotrophic factors (brain-derived neurotrophic factor (BDNF), neurotrophins, glial cell-derived neurotrophic factor (GDNF), etc.) and anti-inflammatory cytokines (IL-4, IL-10, interleukin 13 (IL-13), transforming growth factor beta (TGF-β)) and can contribute to controlling inflammation and improving neuronal survival [7,19].

By using a mouse model of unilateral laser-induced ocular hypertension (OHT), we performed a study comparing the different signs of microglial activation (cell number, soma size, process retraction, number of vertical processes) at different time points (1, 3, 5, 7, and 15 days) after laser application [6,8,9,20,21]. In this work, we observed that the greatest microglial activation, in both the OHT and in the contralateral eyes, occurred three and five days after OHT laser induction, coinciding with high IOP values and with a downregulation of P2RY12 expression in the OHT eyes. P2RY12 is a resident microglial-specific receptor and its expression, which is robust in non-pathological situations, becomes undetectable when there is a strong activation of microglial cells [22]. However, we still do not know the pattern of cytokine release in the OHT eyes and normotensive contralateral eyes at different time points after OHT induction. Thus, in the same unilateral laser-induced OHT experimental model, and also at the same time points after OHT induction, the aim of the current study was to analyze both in OHT and contralateral eyes: (i) the expression of different mediators of inflammation both with neuroprotective and anti-inflammatory properties (IL-4, IL-10, BDNF, VEGF) and with pro-inflammatory properties (TNF-α, IL-1β, IL-6, interleukin 17 (IL-17), IFN-γ), as well as a microglial activation regulator such as fractalkine (CX3CL1), using multiplex assay and immunohistochemical techniques, and (ii) the relationship between the expression of these factors and the decrease in the number of RGCs that occurs after OHT induction.

## 2. Results

### 2.1. Intraocular Pressure

**Figure 1:**
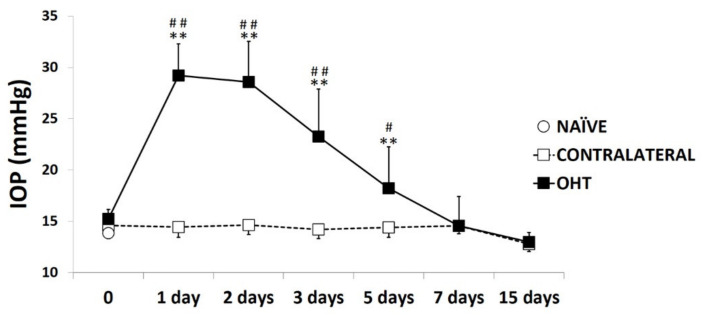
Intraocular pressure (IOP) values at different times after laser-induced ocular hypertension (OHT). Variation over time in intraocular pressure IOP values after OHT induction. Points show the mean levels (±SD) of IOP previously and at 1, 2, 3, 5, 7, and 15 days after laser OHT induction in naïve eyes, ocular hypertension eyes (OHT), and contralateral eyes. Statistical significance indicators: ** *p* < 0.01 vs. naïve; ## *p* < 0.01 vs. contralateral; # *p* < 0.05 vs. contralateral.

In the eyes where OHT was induced, the IOP showed the highest difference with respect to the naïve and contralateral eyes one to three days after laser induction (all: *p* < 0.01; Figure 1). Five days after the OHT induction, the IOP decreased, although it remained significantly higher than in the naïve eyes (*p* < 0.01; Figure 1) and in the contralateral eyes (*p* < 0.05; Figure 1). Seven days after the laser, the IOP in the lasered eyes was similar to that in the naïve and contralateral eyes (Figure 1). At all the time points analyzed, the contralateral eyes had an IOP similar to that of the naïve eyes (*p* > 0.05; Figure 1).

### 2.2. Levels of Pro-Inflammatory Mediators in the Course of Experimental Glaucoma

#### 2.2.1. Interleukin 1 Beta (IL-1β)

**Figure 2:**
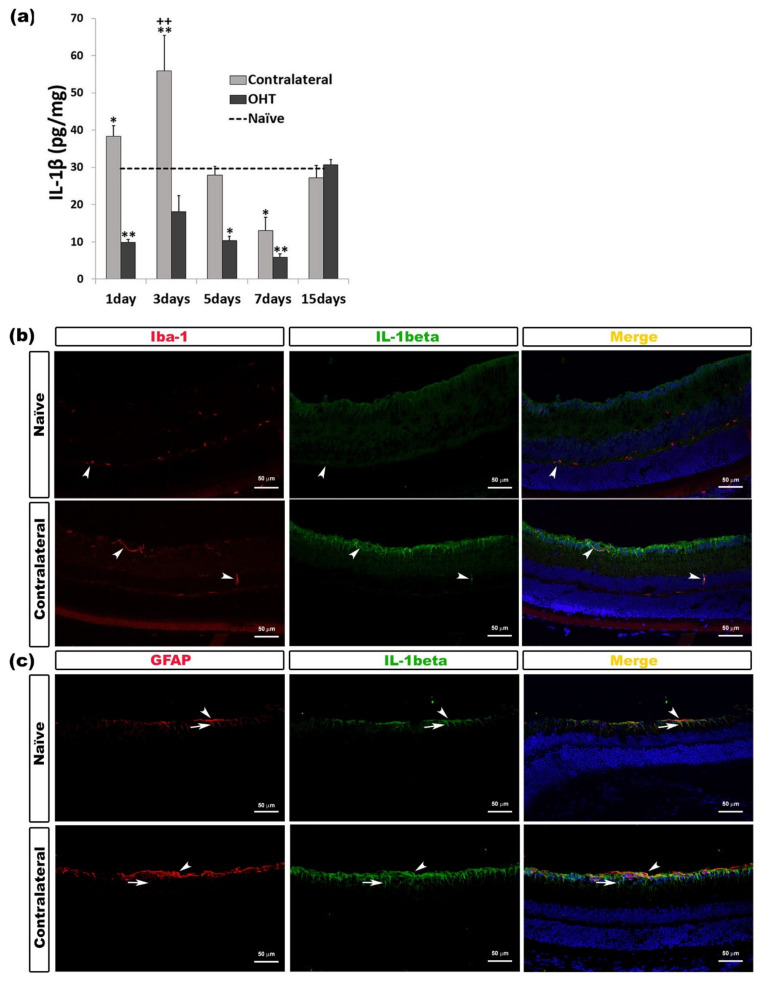
IL-1β levels at different times after laser-induced ocular hypertension (OHT). (**a**) The variation over time in the IL-1β levels in the multiplex assay. The histogram shows the mean levels (±SD) of IL-1β (pg/mg) at days 1, 3, 5, 7, and 15 after laser OHT induction, in ocular hypertension eyes (OHT) and in contralateral eyes. The dashed line indicates the mean levels in naïve eyes. Statistical significance indicators: * *p* < 0.05, ** *p* < 0.01 vs. naïve; ++ *p* < 0.01 vs. OHT. (**b**,**c**) Immunohistochemical study of IL-1β expression in naïve and contralateral eyes three days after unilateral laser-induced OHT. Retinal sections were immunolabeled with antibodies to IL-1β (green), Iba-1 (red in (**b**)), or GFAP (red in (**c**)). Merge (yellow). (**b**) The arrowheads show the co-expression of Iba-1 and IL-1β. This expression is higher in contralateral eyes than in naïve eyes. (**c**) Arrowheads (astrocytes) and arrows (Müller cells) indicate the co-expression of GFAP and IL-1β, with the expression being more intense in contralateral than in naïve eyes. Abbreviations: OHT (ocular hypertension); IL-1β (interleukin 1 beta); GFAP (glial fibrillary acidic protein).

IL-1β overexpression was only detected in the contralateral eyes. After OHT induction, IL-1β expression in the contralateral eyes increased significantly after one day (*p* < 0.05) and three days (*p* < 0.01). However, after seven days, contralateral eyes showed a significant decrease (*p* < 0.01) in comparison with naïve eyes. In the OHT eyes, IL-1β expression was significantly lower than in naïve eyes after one day (*p* < 0.01), five days (*p* < 0.05), and seven days (*p* < 0.01). After three days, in contralateral eyes, the IL-1β expression was significantly higher (*p* < 0.01) than in OHT eyes (Figure 2a). The immunohistochemical techniques showed that IL-1β was expressed by microglia (Figure 2b) and macroglia (astrocytes and Müller cells) (Figure 2c) (Table 1).

#### 2.2.2. Interleukin 6 (IL-6)

**Figure 3:**
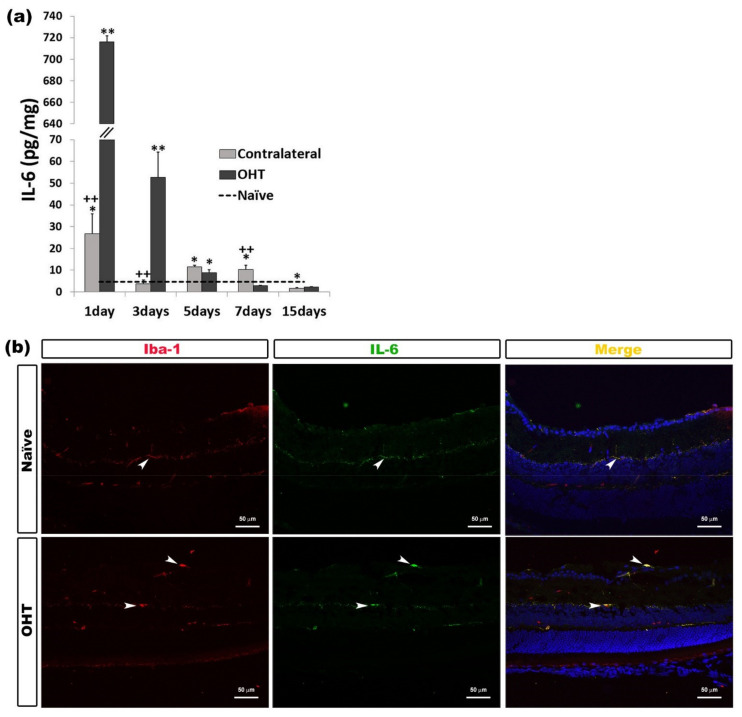
IL-6 levels at different times after laser-induced ocular hypertension (OHT). (**a**) The variation over time in the IL-6 levels in the multiplex assay. The histogram shows the mean levels (±SD) of IL-6 (pg/mg) at days 1, 3, 5, 7, and 15 after laser OHT induction, in ocular hypertension eyes (OHT) and in contralateral eyes. The dashed line indicates the mean levels in naïve eyes. Statistical significance indicators: * *p* < 0.05, ** *p* < 0.01 vs. naïve; ++ *p* < 0.01 vs. OHT. (**b**) Immunohistochemical study of IL-6 expression in OHT eyes one day after unilateral laser-induced OHT. Retinal sections were immunolabeled with antibodies to IL-6 (green) and Iba-1 (red). Merge (yellow). The arrowheads show the co-expression of Iba-1 and IL-6 in OHT and naïve eyes. This expression is higher in OHT eyes than in naïve eyes. Abbreviations: OHT (ocular hypertension); IL-6 (interleukin 6); Iba-1 (ionized calcium-binding adaptor molecule 1). Values at different times after laser-induced ocular hypertension (OHT). Variation over time in intraocular pressure IOP values after OHT induction. IL-1β levels at different times after laser-induced ocular hypertension (OHT).

In OHT eyes, a significant increase in IL-6 expression was observed at one and three days (*p* < 0.01), and five days (*p* < 0.05) compared with naïve eyes. In the contralateral eyes, this significant increase was detected at days 1, 5, and 7 (all: *p* < 0.05) after laser induction; however, at day 15, a significant decrease (*p* < 0.05) was observed with respect to naïve eyes. In contralateral eyes, with respect to OHT eyes, the IL-6 expression was significantly lower after one and three days (*p* < 0.01) and significantly greater after seven days (*p* < 0.01) (Figure 3a). The immunohistochemical techniques revealed that IL-6 was expressed by microglia (Figure 3b) (Table 1).

#### 2.2.3. Interferon Gamma (INF-γ)

**Figure 4:**
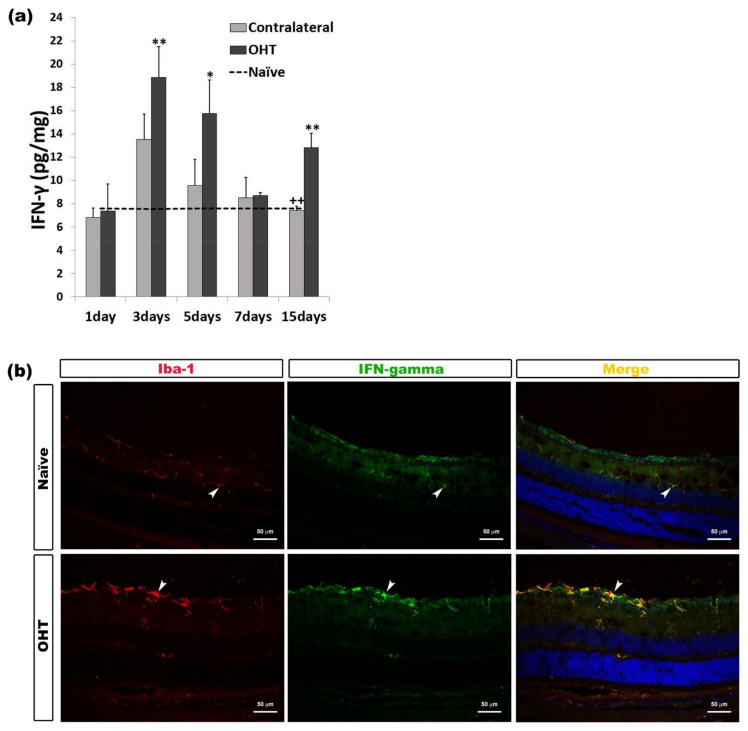
IFN-γ levels at different times after laser-induced ocular hypertension (OHT). (**a**) The variation over time in the IFN-γ levels in the multiplex assay. The histogram shows the mean levels (±standard deviation, SD) of IFN-γ (pg/mg) at days 1, 3, 5, 7, and 15 after laser OHT induction, in ocular hypertension eyes (OHT) and in contralateral eyes. The dashed line indicates the mean levels in naïve eyes. Statistical significance indicators: * *p* < 0.05, ** *p* < 0.01 vs. naïve; ++ *p* < 0.01 vs. OHT. (**b**) Immunohistochemical study of IFN-γ expression in OHT eyes three days after unilateral laser-induced OHT. Retinal sections were immunolabeled with antibodies to IFN-γ (green) and Iba-1 (red). Merge (yellow). The arrowheads indicate the co-expression of Iba-1 and IFN-γ in both naïve and OHT eyes. The expression is more intense in OHT eyes. Abbreviations: OHT (ocular hypertension); IFN-γ (interferon-gamma); Iba-1 (ionized calcium-binding adaptor molecule 1).

In comparison with naïve eyes, the expression of INF-γ in OHT eyes showed a significant increase at days 3 (*p* < 0.01), 5 (*p* < 0.05), and 15 (*p* < 0.01) after laser induction. No difference was observed between contralateral and naïve eyes. The comparison between contralateral and OHT eyes showed that the INF-γ expression in contralateral eyes was significantly lower after 15 days (*p* < 0.01) (Figure 4a). In the histological sections, it was observed that the immunolabeling of INF-γ was related to microglial cells (Figure 4b) (Table 1).

#### 2.2.4. Interleukin 17 (IL-17)

**Figure 5:**
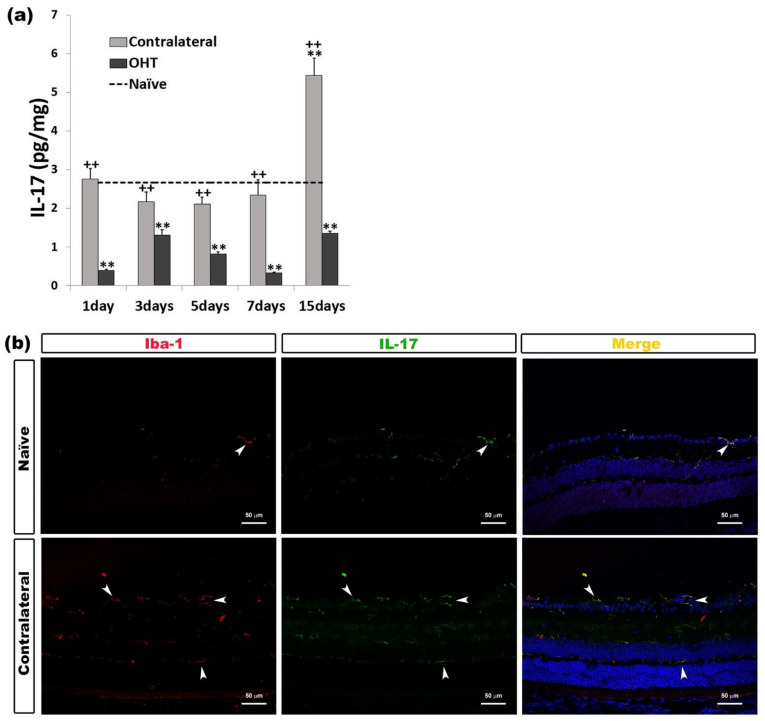
IL-17 levels at different times after laser-induced ocular hypertension (OHT). (**a**) The variation over time in the IL-17 levels in the multiplex assay. The histogram shows the mean levels (±SD) of IL-17 (pg/mg) at days 1, 3, 5, 7, and 15 after laser OHT induction, in ocular hypertension eyes (OHT) and in contralateral eyes. The dashed line indicates the mean levels in naïve eyes. Statistical significance indicators: ** *p* < 0.01 vs. naïve; ++ *p* < 0.01 vs. OHT. (**b**) Immunohistochemical study of IL-17 expression in contralateral eyes 15 days after unilateral laser-induced OHT. Retinal sections were immunolabeled with antibodies to IL-17 (green) and Iba-1 (red). Merge (yellow). The arrowheads show the co-expression of Iba-1 and IL-17 in naïve and contralateral eyes; it is more intense in contralateral eyes. Abbreviations: OHT (ocular hypertension); IL-17 (interleukin 17); Iba-1 (ionized calcium-binding adaptor molecule 1).

Only after 15 days, in contralateral eyes, we observed a significant increase in the IL-17 expression (*p* < 0.01) with respect to naïve eyes; however, this expression was significantly greater than in OHT eyes at all time points analyzed (all: *p* < 0.01). In the OHT eyes, the IL-17 expression was significantly lower than in naïve eyes at all time points analyzed (all: *p* < 0.01) (Figure 5a). The immunohistochemical techniques showed that IL-17 was expressed by microglial cells (Figure 5b) (Table 1).

#### 2.2.5. Tumor Necrosis Factor Alpha (TNF-α)

**Figure 6:**
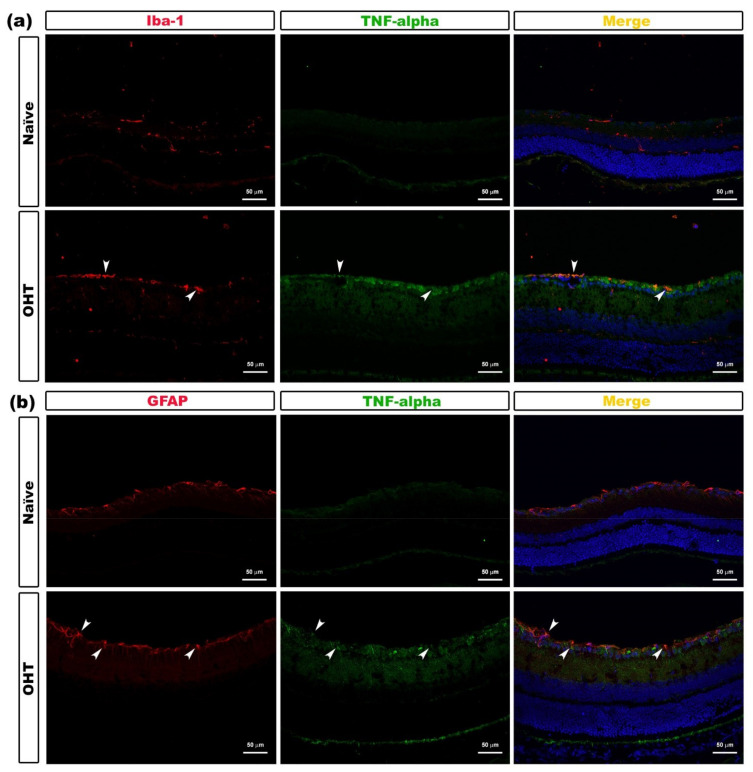
TNF-α expression in OHT eyes after laser-induced ocular hypertension (OHT). (**a**,**b**) Immunohistochemical study of TNF-α expression in OHT eyes at 1 day after unilateral laser-induced OHT. Retinal sections were immunolabeled with antibodies to TNF-α (green), Iba-1 (red in (**a**)), and GFAP (red in (**b**)). Merge (yellow). (**a**) Arrowheads point to the co-expression of Iba-1 and TNF-α in OHT eyes. (**b**) Arrowheads show the co-expression of GFAP and TNF-α in the OHT eyes. Abbreviations: OHT (ocular hypertension); TNF-α (tumor necrosis factor-alpha); GFAP (glial fibrillary acidic protein); Iba-1 (ionized calcium-binding adaptor molecule 1).

With respect to the TNF-α expression in this work, a detectable concentration level of this cytokine was not obtained, but the immunohistochemical analysis showed that TNF-α expression was localized in the microglial cells (Figure 6a) and astrocytes (Figure 6b) (Table 1).

### 2.3. Levels of Anti-Inflammatory Mediators in the Course of Experimental Glaucoma

#### 2.3.1. Interleukin 4 (IL-4)

**Figure 7:**
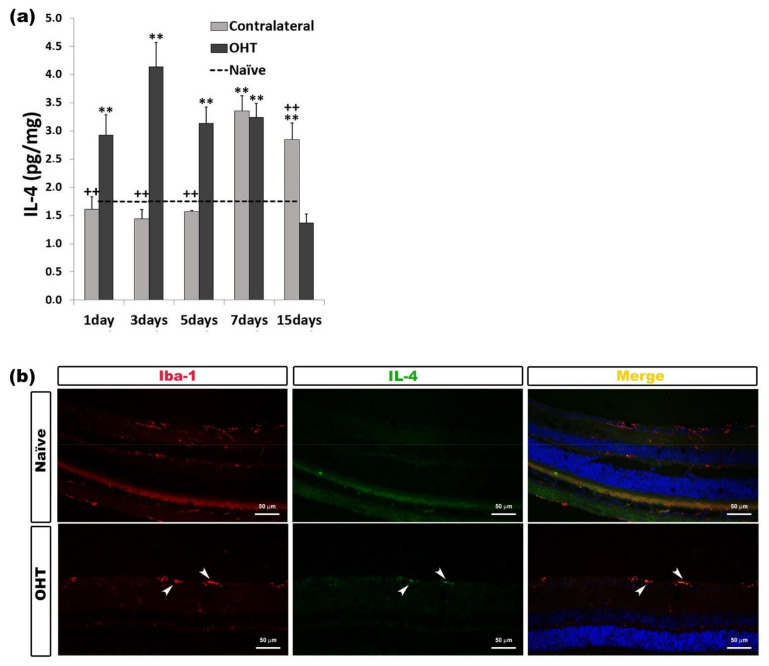
IL-4 levels at different times after laser-induced ocular hypertension (OHT). (**a**) Variation over time in the IL-4 levels in the multiplex assay. The histogram shows the mean levels (±SD) of IL-4 (pg/mg) at days 1, 3, 5, 7, and 15 after laser OHT induction, in ocular hypertension eyes (OHT) and in contralateral eyes. The dashed line indicates the mean levels in naïve eyes. Statistical significance indicators: ** *p* < 0.01 vs. naïve; ++ *p* < 0.01 vs. OHT. (**b**) Immunohistochemical study of IL-4 expression in OHT eyes three days after unilateral laser-induced OHT. Retinal sections were immunolabeled with antibodies to IL-4 (green) and Iba-1 (red). Merge (yellow). The arrowheads indicate the co-expression of Iba-1 and IL-4 in OHT eyes. Abbreviations: OHT (ocular hypertension); IL-4 (interleukin 4); Iba-1 (ionized calcium-binding adaptor molecule 1).

In comparison with the naïve group, in the OHT eyes, we observed a significant increase in the IL-4 expression at days 1, 3, 5, and 7 (all: *p* < 0.01) after laser photocoagulation. However, in the contralateral eyes, this significant increase was observed at days 7 and 15 (*p* < 0.01) after laser induction. The comparison between OHT eyes and contralateral eyes showed that, in contralateral eyes, the IL-4 expression was significantly lower (*p* < 0.01) at days 1, 3, and 5 and significantly higher at day 15 (*p* < 0.01) than in OHT eyes. (Figure 7a). In the immunohistochemical sections, IL-4 was expressed by microglial cells (Figure 7b) (Table 1).

#### 2.3.2. Interleukin 10 (IL-10)

**Figure 8:**
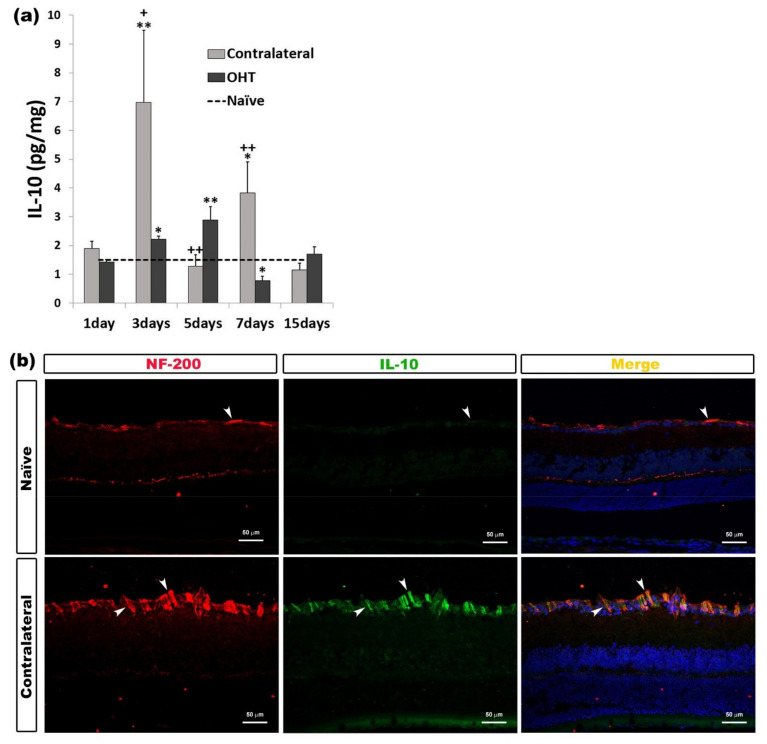
IL-10 levels at different times after laser-induced ocular hypertension (OHT). (**a**) The variation over time in the IL-10 levels in the multiplex assay. The histogram shows the mean levels (±SD) of IL-10 (pg/mg) at days 1, 3, 5, 7, and 15 after laser OHT induction, in ocular hypertension eyes (OHT) and in contralateral eyes. The dashed line indicates the mean levels in naïve eyes. Statistical significance indicators: * *p* < 0.05, ** *p* < 0.01 vs. naïve; + *p* < 0.05, ++ *p* < 0.01 vs. OHT. (**b**) Immunohistochemical study of IL-10 expression in contralateral eyes three days after unilateral laser-induced OHT. Retinal sections were immunolabeled with antibodies to IL-10 (green) and NF-200 (red). Merge (yellow). The arrowheads indicate the co-expression of NF-200 (retinal ganglion cell (RGC) axons) and IL-10 in naïve and contralateral eyes; it is more intense in contralateral eyes. Abbreviations: OHT (ocular hypertension); IL-10 (interleukin 10); NF-200 (neurofilament of 200 KDa).

In OHT eyes, we observed a significant increase after three (*p* < 0.05) and five days (*p* < 0.01), while seven days after OHT induction, the IL-10 expression was significantly lower (*p* < 0.05) than in naïve eyes. In contralateral eyes, the IL-10 expression was significantly higher after three (*p* < 0.01) and seven days (*p* < 0.05) with respect to naïve eyes. Compared to OHT eyes, contralateral eyes showed a significant increase in IL-10 expression after three (*p* < 0.05) and seven days (*p* < 0.01), with this expression being significantly less after five days (*p* < 0.01) (Figure 8a). Immunohistochemical techniques showed that IL-10 expression colocalized with the NF-200+ axons of the RGCs (Figure 8b) (Table 1).

#### 2.3.3. Brain-Derived Neurotrophic Factor (BDNF)

**Figure 9:**
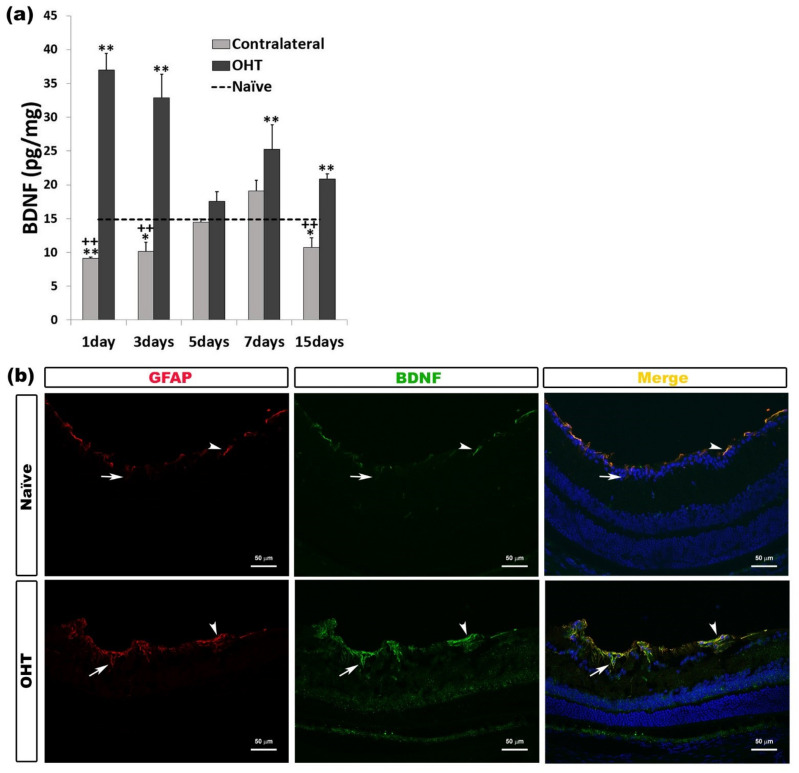
Brain-Derived Neurotrophic Factor (BDNF) levels at different times after laser-induced ocular hypertension (OHT). (**a**) The variation over time in the BDNF levels in the multiplex assay. The histogram shows the mean levels (±SD) of BDNF (pg/mg) at days 1, 3, 5, 7, and 15 after laser OHT induction, in ocular hypertension eyes (OHT) and in contralateral eyes. The dashed line indicates the mean levels in naïve eyes. Statistical significance indicators: * *p* < 0.05, ** *p* < 0.01 vs. naïve; ++ *p* < 0.01 vs. OHT. (**b**) Immunohistochemical study of BDNF expression in OHT eyes one day after unilateral laser-induced OHT. Retinal sections were immunolabeled with antibodies to BDNF (green) and GFAP (red). Merge (yellow). The arrowheads (astrocytes) and arrows (Müller cells) indicate the co-expression of GFAP and BDNF, with the expression being more intense in OHT than in naïve eyes. Abbreviations: OHT (ocular hypertension); BDNF (brain-derived neurotrophic factor); GFAP (glial fibrillary acidic protein).

In OHT eyes, we detected a significant increase in the BDNF expression at all time points analyzed except five days (all: *p* < 0.01). In contralateral eyes, BDNF expression was significantly lower than in naïve eyes after 1 day (*p* < 0.01), and 3 and 15 days (*p* < 0.05). Contralateral eyes showed significantly lower BDNF expression compared to OHT eyes at days 1, 3, and 15 (all: *p* < 0.01) (Figure 9a). Immunohistochemical analysis showed that the BDNF expression was located in the macroglial cells (astrocytes and Müller cells) (Figure 9b) (Table 1).

#### 2.3.4. Vascular Endothelial Growth Factor (VEGF)

**Figure 10:**
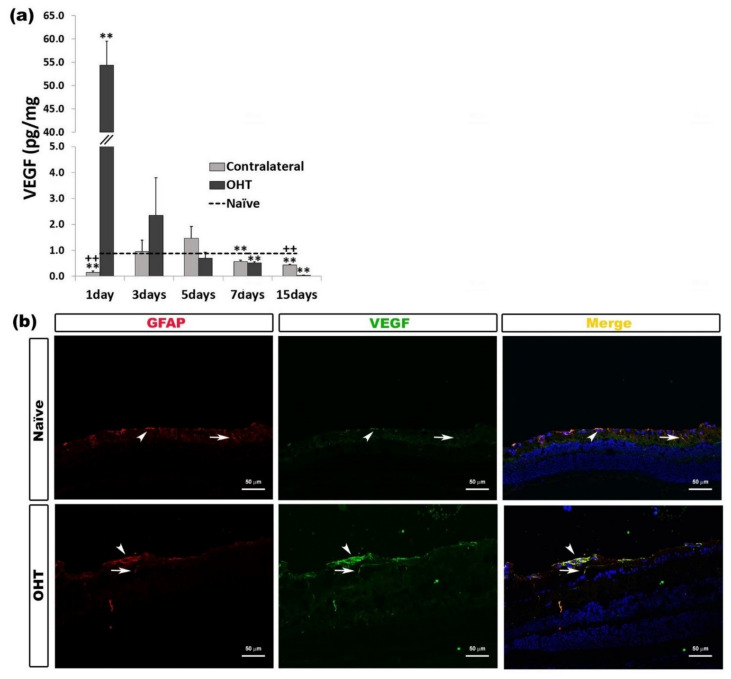
Vascular Endothelial Growth Factor (VEGF) levels at different times after laser-induced ocular hypertension (OHT). (**a**) The variation over time in the VEGF levels in the multiplex assay. The histogram shows the mean levels (±SD) of VEGF (pg/mg) at days 1, 3, 5, 7, and 15 after laser OHT induction, in ocular hypertension eyes (OHT) and in contralateral eyes. The dashed line indicates the mean levels in naïve eyes. Statistical significance indicators: ** *p* < 0.01 vs. naïve; ++ *p* < 0.01 vs. OHT. (**b**) Immunohistochemical study of VEGF expression in OHT eyes one day after unilateral laser-induced OHT. Retinal sections were immunolabeled with antibodies to VEGF (green) and GFAP (red). Merge (yellow). The arrowheads (astrocytes) and arrows (Müller cells) show the co-expression of GFAP and VEGF, with the expression being higher in OHT than in naïve eyes. Abbreviations: OHT (ocular hypertension); VEGF (vascular endothelial growth factor); GFAP (glial fibrillary acidic protein).

One day after laser induction, VEGF expression in OHT eyes was significantly higher (*p* < 0.01), and in contralateral eyes, it was significantly lower (*p* < 0.01), both in comparison with naïve eyes. This VEGF expression in OHT eyes and in contralateral eyes was significantly lower at days 7 and 15 (*p* < 0.01) than in naïve eyes. In addition, in comparison with OHT eyes, contralateral eyes showed a significant decrease in VEGF expression after one day (*p* < 0.01), with this expression being significantly greater 15 days (*p* < 0.01) after laser induction (Figure 10a). The immunohistochemical techniques showed that VEGF was expressed by macroglia (astrocytes and Müller cells) (Figure 10b) (Table 1).

### 2.4. Levels of Microglial Activation Regulator in the Course of Experimental Glaucoma

#### CX3CL1 (Fractalkine)

In the OHT retinas, we observed a significant increase in fractalkine expression after one day (*p* < 0.01) in comparison with naïve eyes. However, this expression of fractalkine in OHT eyes was significantly lower than in naïve eyes at all the other time points analyzed (days 3, 5, and 7, *p* < 0.01; 15 days, *p* < 0.05). The contralateral eyes showed a significant decrease in fractalkine expression at days 1, 3, and 5 (all: *p* < 0.01) compared to naïve eyes.

**Figure 11:**
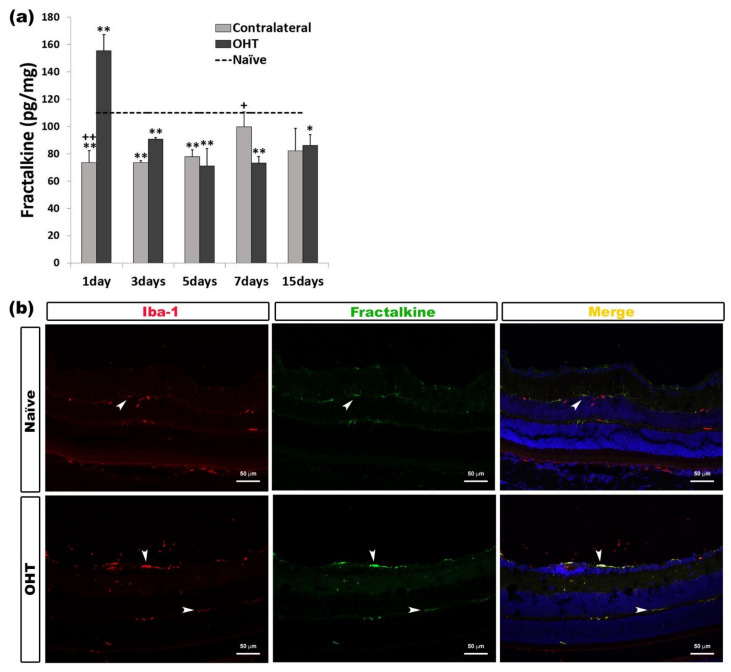
Fractalkine levels at different times after laser-induced ocular hypertension (OHT). (**a**) The variation over time in the fractalkine levels in the multiplex assay. The histograms show the mean levels (±SD) of fractalkine (pg/mg) at days 1, 3, 5, 7, and 15 after laser OHT induction, in ocular hypertension eyes (OHT) and in contralateral eyes. The dashed line indicates the mean levels in naïve eyes. Statistical significance indicators: * *p* < 0.05, ** *p* < 0.01 vs. naïve; + *p* < 0.05, ++ *p* < 0.01 vs. OHT. (**b**) Immunohistochemical study of fractalkine expression in OHT eyes one day after unilateral laser-induced OHT. Retinal sections were immunolabeled with antibodies to fractalkine (green) and Iba-1 (red). Merge (yellow). The arrowheads indicate the co-expression of Iba-1 and fractalkine in OHT eyes and naïve eyes; it is more intense in OHT eyes. Abbreviations: OHT (ocular hypertension); Iba-1 (ionized calcium-binding adaptor molecule 1).

In addition, in contralateral eyes, the expression of fractalkine was significantly lower than in OHT eyes after one day (*p* < 0.01) and significantly greater after seven days (*p* < 0.05) than in OHT eyes (Figure 11a). Fractalkine expression was observed in microglial cells when the processed sections were analyzed with immunohistochemical techniques (Figure 11b) (Table 1).

### 2.5. Number of Brn3a+ RGCs in the Course of Experimental Glaucoma

#### 2.5.1. Temporal Profile

**Figure 12:**
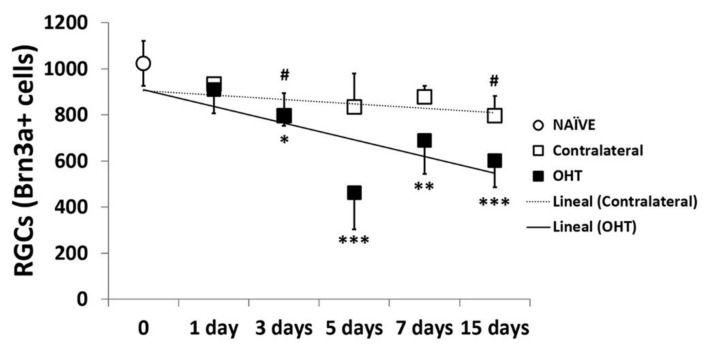
The variation over time in the number of Brn3a+ cells in the immunohistochemical assay. Points show the mean levels (±SD) of Brn3a+ cells at days 1, 3, 5, 7, and 15 after laser OHT induction in control naïve eyes, ocular hypertension eyes (OHT), and in contralateral eyes. Statistical significance indicators: * *p* < 0.05, ** *p* < 0.01, *** *p* < 0.005, OHT vs. control naïve; # *p* < 0.05, contralateral vs. control naïve. Abbreviations: RGCs (retinal ganglion cells); Brn3a (brain-specific homeobox/POU domain protein 3A); OHT (ocular hypertension).

In OHT eyes, a significant reduction in the number of Brn3a+ cells was observed three days after laser induction (*p* < 0.05), and the decrease became more severe at days 5, 7, and 15 (*p* < 0.005, *p* < 0.01, and *p* = 0.005, respectively) compared with control naïve eyes. In the contralateral eyes, a modest decrease was also observed, but it only achieved statistical significance after 3 and 15 days (*p* < 0.05) with respect to naïve eyes (Figure 12).

#### 2.5.2. Spatial Distribution

**Figure 13:**
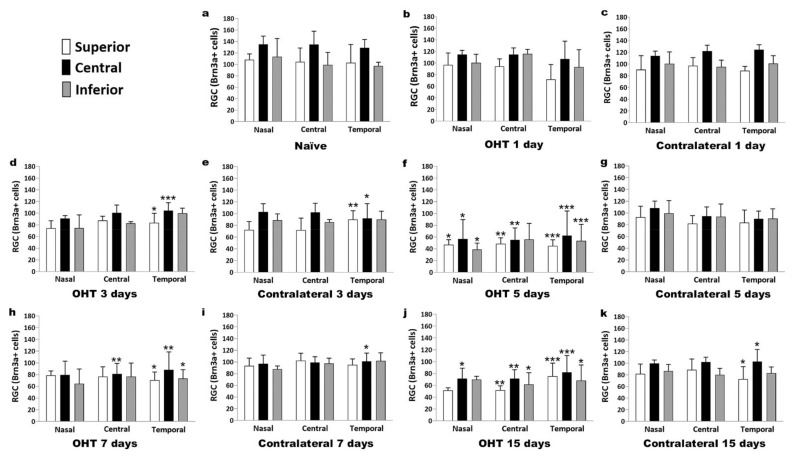
The variation over space and time in the number of Brn3a+ cells in the immunohistochemical assay. The histograms show the mean levels (±SD) of Brn3a+ cells at days 1, 3, 5, 7, and 15 after laser OHT induction. (**a**) Control naïve eyes; (**b**,**d**,**f**,**h**,**j**) ocular hypertension eyes (OHT); (**c**,**e**,**g**,**i**,**k**) contralateral eyes. In each region and zone of the retina: nasal‒superior, nasal‒central, nasal‒inferior; central‒superior, central‒central, central‒inferior; and temporal‒superior, temporal‒central, temporal‒inferior. Statistical significance indicators: * *p* < 0.05, ** *p* < 0.01, *** *p* < 0.005, OHT vs. control naïve. Abbreviations: RGCs (retinal ganglion cells); Brn3a (brain-specific homeobox/POU domain protein 3A); OHT (ocular hypertension).

In OHT eyes, a decrease in the number of Brn3a+ RGCs was initiated at day 3 (Figure 13d) in the temporal region, and specifically in its central zone; at day 5 (Figure 13f), an extensive decrease in the number of Brn3a+ RGCs was observed, with the temporal region and all its zones being more dramatically involved. The central region and its central and superior zones also showed significant decreases, while the nasal region recorded the lowest reduction in the number of Brn3a+ RGCs. At days 7 (Figure 13h) and 15 (Figure 13j), the decrease in Brn3a+ RGCs was still significant in the temporal and central retinal regions. These changes seemed to be less evident in the inferior retina. In the contralateral eyes, the decrease in the number of Brn3a+ RGCs was also initiated at day 3 (Figure 13e) in the temporal region, but its superior and central zones were the only ones that underwent changes. The decrease in the number of Brn3a+ RGCs was significant at day 7 (Figure 13i), but only in the temporal central retina, and at day 15 (Figure 13k) in the temporal central and temporal nasal retina.

## 3. Discussion

To our knowledge, this is the first work that has analyzed the temporal pattern of expression of pro-inflammatory cytokines (IL-1β, IFNγ, TNF-α, IL-6, and IL-17), anti-inflammatory cytokines (IL-4, IL-10), neurotrophic factors with neuroprotective and anti-inflammatory properties (BDNF, VEGF), and fractalkine (CX3CL), a microglial activation regulator, in a mouse experimental model of unilateral laser-induced OHT.

Chronic microglial activation has been shown to be one of the main pathogenic mechanisms involved in the development of neurodegenerative diseases, including glaucoma [7,10]. Activated microglial cells can adopt two phenotypes in response to damage. The M1-like or neurotoxic phenotype generates a large inflammatory response, releasing pro-inflammatory cytokines and reactive oxygen and nitric oxide species. Initially, this response could be protective in an attempt to restore homeostasis of nerve tissue. However, a chronic activation of M1-like can induce neurodegeneration due to the excessive release of neurotoxic molecules and pro-inflammatory cytokines [23,24,25]. Alternatively, the M2-type phenotype releases neurotrophic factors and anti-inflammatory mediators that have the ability to moderate the pro-inflammatory response and promote tissue repair, leading to a supportive neuronal environment [12,26,27]. Among the inflammation mediators, IFN-γ, TNF-α, IL-1β, IL-6, IL-12, IL-17, and IL-18 are well-known pro-inflammatory cytokines, whereas IL4, IL-10, IL-13, and transforming growth factor-beta (TGF-β) are recognized as anti-inflammatory cytokines.

Our study showed a significant increase in the expression of inflammation mediator IFN-γ at days 3, 5, and 15 in the OHT eyes with respect to the naïve ones after the laser OHT induction. In the contralateral eyes, although the values were also elevated (at days 3, 5, and 7) with respect to the naïve eyes, these changes were not statistically significant. In addition, in our immunohistochemical sections, the immunolabeling of IFN-γ was related to the microglial cells. IFN-γ is one of the most efficient natural inductors of microglial activation [28]. This molecule promotes the upregulation of MHCI and MHCII, the production of cytokines, and the induction of components of the complement cascade. Microglia polarize the phenotype M1 when stimulated with IFN-γ and can produce inflammatory mediators such as IL-1β, IL-6, TNF-α, C-C chemokine ligand 2 (CCL2), reactive oxygen species (ROS), and nitric oxide (NO) [29]. Thus, this cytokine is important in amplifying the effects of other cytokines and growth factors, playing a key role in maintaining and extending the immune response [30]. The data on this cytokine in the present study coincide with those found in our previous studies of OHT eyes [21], in which we found a greater activation of the microglial cells at days 3 and 5 and a downregulation in the expression of P2RY12 in these cells, indicating a greater inflammatory process. In the contralateral eyes, at these times (days 3 and 5), we have also shown greater microglial activation, although to a lesser degree than in the OHT eyes [21], which would coincide with the non-significant increase in this cytokine.

In the present study, we found a significant decrease in the expression of IL-1β in OHT eyes at days 1, 5, and 7 in comparison to naïve eyes. However, in the contralateral eyes, we found a significant increase in IL-1β at days 1 and 3 and a significant decrease at day 7, being expressed by both microglia and macroglia, as we could observe in our immunohistochemical study. These results seem to be contradictory to previous results from other models. In hypoxia conditions, amoeboid microglial cells increased the production of TNF-α and IL-1β, suggesting that the binding of the cytokines to their respective receptors could be one of the most important factors related to RCG death [31]. IL-1β was significantly elevated in both proximal and distal portions of the optic nerve in DBA/2J mice compared to controls, indicating a possible role for this cytokine in distal axonopathy in glaucoma [32]. In a unilateral laser-induced OHT model in rats, a significant increase in IL-1β protein levels was observed at days 3 and 7 after OHT induction compared to control animals [33]. However, Chidlow et al. [34], in an experimental glaucoma model by laser photocoagulation of the trabecular meshwork in rats, did not find TNF-α and IL-1β immunolabeling in either the retina or the optic nerve, despite evidence of moderate IL-1β and TNF-α mRNA upregulation in response to elevated IOP. The authors proposed three possible explanations for this: (i) the mRNAs were not translated; (ii) the proteins were only transiently expressed by individual cells or degraded rapidly, which would make immunohistochemical detection more difficult; and (iii) the tissue level of the proteins was below the detectable limit of the relevant assays. In our study, we also could not detect TNFα with the multiplex assay, and we believe that Chidlow et al.’s third explanation could support this fact. However, we were able to obtain immunolabeling of TNFα on microglial cells and macroglial cells. It has been shown that both types of retinal cells can express this cytokine in glaucoma [35].

Contrary to what was observed in OHT eyes, we were able to detect an upregulation of IL-1β in the contralateral eye. The downregulation of IL1- β at days 1, 3, 5, and 7 in OHT eyes could be explained by the action of IL4 and IL10. These cytokines may inhibit the production of IL-1β and TNF-α by monocyte-derived macrophages and may be important in controlling the immune response by negatively regulating the production of pro-inflammatory mediators [36,37]. In our study, the significant upregulation of IL-4 (at days 1, 3, 5, and 7) and IL-10 (at days 3 and 5) would coincide with the significant downregulation of IL-1 β (at days 1, 5, and 7) in OHT eyes. In addition, in the contralateral eyes, upregulation of IL-1β (at days 1 and 3) and downregulation at day 7 would coincide with downregulation of IL4 (at days 3 and 5) and upregulation at day 7. In our study, IL-4 was detected immunohistochemically in microglial cells, while IL-10 was located in the axons of the RGCs. It has been observed that RGC cultures express IL-10 receptors, suggesting that this cytokine may have a direct neuroprotective effect on RGC axons [38]. The anti-inflammatory cytokines IL-4 and IL-10 may play a compensatory role early in glaucoma, prior to functional transport loss and RGC death [39], and could induce alternative activation and polarization of M2 microglia [29].

In our work, we found a significant increase in IL-6 in OHT eyes at days 1, 3, and 5 after OHT induction with respect to naïve eyes, being expressed by microglial cells, as we observed in our immunohistochemical study. There is substantial evidence of a strong relationship between IL-6 secretion and increased IOP. Johnson et al. [40] found early upregulation of IL-6 mRNA in the optic nerve head as an early response to high IOP levels in a hypertonic saline OHT rat model. In an OHT experimental model of laser photocoagulation in rats [34], researchers found a statistically significant upregulation of IL-6 expression in the retinas using ELISA techniques at days 1, 3, and 7 after induction of elevated IOP, with the highest value at day 1. It has been found that the expression of IL-6 may be key to the microglial response to elevated pressure. In RGC cultures subjected to high hydrostatic pressure, a decrease in cell survival occurs when the IL-6 supplied by the microglia decreases, suggesting that IL-6 released by the microglia may be counteracting proapoptotic factors such as TNF-α, nitric oxide, and other factors [41]. In our study, we found that in OHT eyes, the major expression of IL-6 coincides with the times when the IOP is highest (days 1, 3, and 5). We agree that there could be a close relationship between an elevation of IOP and an increase in IL-6, as stated by other authors [34,40]. Moreover, in our study, we observed that when the IOP normalizes (seven days after OHT induction), IL-6 decreases, with no significant difference being seen with respect to naïve eyes. This coincides with the fact that after seven days, there is a significant death of the RGCs in this experimental model [42,43,44]. In addition, in our study, three days after the induction of OHT, there was a decrease in Brn3a+ RGC, and this decrease increased at subsequent time points, which was parallel to the decrease in the secretion of IL-6. Therefore, this would support the notion that IL-6 exerts a neuroprotective effect, counteracting the proapoptotic effects of other factors and induced by the increase in IOP, as has been reported by other authors [41]. In our study, after laser induction, we also observed an increase in IL-6 expression (at days 1, 5, and 7) in normotensive contralateral eyes with respect to naïve eyes. Therefore, the increase in IL-6 in the contralateral eyes, which was less than in OHT eyes, could be related to factors other than the increase in IOP. In previous studies, we have shown that an activation of microglial cells in the contralateral eyes is taking place that could be caused by immune signals derived from the OHT eyes [6,8,9,20,45]. Microglia have been shown to be the main retinal cell type responsible for IL-6 synthesis after inflammatory damage [34]. It has been shown that IL-6 pretreatment can prevent apoptosis of neuronal cells exposed to different physiological stressors in vitro, which would support the idea that IL-6 may have neuroprotective effects [46].

IL-17 is an inflammatory response-inducing cytokine, as it can induce other cytokines and inflammatory chemokines [47]. Astrocytes [48], infiltrated T cells [49], and microglia [50] may be responsible for secreting this cytokine. Surprisingly, in our study, we only found a significant increase in IL-17 in the contralateral eyes 15 days after laser induction, and we also observed that it was expressed by microglial cells. However, in OHT eyes, we found a statistically significant decrease in IL-17 with respect to naïve eyes at all time points analyzed. A possible explanation for this could be that cytokines with an anti-inflammatory character could be counteracting the action of IL-17 in OHT eyes, as IL-10 and IL-4 did on IL-1β. It has been shown that several cytokines such as IL-4, IL10, and IL-13 may have an inhibitory effect on IL-17 [51,52]. A possible explanation for the high IL-17 levels in the contralateral eyes could be that IL-1β is elevated in these eyes. It has been shown that IL-17 is produced by the microglia in response to IL-1β [50]. In addition, IL-17 may have an anti-inflammatory and neuroprotective effect that is produced by astrocytes under short-term stimulation of a high-level IL-17 cytokine in the culture medium [53,54]. This effect may be occurring in the contralateral eyes 15 days after OHT induction: a significant increase in this cytokine was observed only at this time point.

Microglia express the chemokine fractalkine receptor (CX3CR1), and their signaling could regulate microglial behavior in glaucoma. Fractalkine (CX3CL1) is constitutionally expressed by the neurons and endothelial cells of the retina and is upregulated in conditions of inflammation [55,56]. In our study, we observed in the OHT eyes a significant increase in fractalkine on day 1 after OHT induction with respect to naive eyes and a positive immunolabeling of this chemokine on the microglial cells. This increase was not observed at later time points after OHT laser induction. It has been suggested that fractalkine released by apoptotic neurons may act as a “find me” signal promoting microglial processes to be directed towards apoptotic neurons for elimination [57]. In a previous study, we found that microglial migrations, the reorientation of processes towards certain areas of the retina, and the appearance of amoeboid microglia occurred one day after OHT induction [20,21]. Therefore, the fractalkine released by RGCs could promote the activation and migration of microglial cells observed at an early time, such as day 1, after OHT laser induction. In addition, fractalkine signaling regulates cytokine secretion, phagocytosis, the migration and survival of circulating monocytes, and recruitment of chemokine C-C motif receptor 2 (Ccr2)-positive monocytes [56,58,59,60,61]. In previous works, one day after OHT induction, we also found that in the OHT eyes, there was a large number of rounded MHC-II+ cells near vascular zones and that they were phagocyted by the amoeboid microglia [20,21]. These rounded cells could be macrophages and monocytes that could have infiltrated the retinal tissue, helped by the increase in fractalkine in the tissue. In addition, rounded MHC-II+ cells could have entered the retinal tissue due to an alteration of the blood‒retinal barrier in the OHT eye [20].

In the present study, we observed that, one day after OHT induction, there is a significant VEGF upregulation, not found at later time points after OHT laser induction or in the contralateral eyes. It has been demonstrated that VEGF can induce increased vascular permeability and is a chemoattractant of monocytes/macrophages (which have VEGF receptors), allowing them to migrate into the tissues [62]. In the retina, VEGF is principally expressed in endothelial cells [63], the retinal pigment epithelium [64], Müller cells [65], astrocytes [66], and RGCs [67]. In our immunohistochemical study, we found the expression of this cytokine in the macroglial cells. This vascular factor has been suggested as a survival factor for retinal neurons [68]. The cause of an elevated VEGF concentration in eyes with glaucoma may be related to the ischemia, hypoxia, or elevated reactive oxygen intermediates caused by glaucomatous damage [17].

Another factor that could have a protective effect against the neurodegeneration produced by OHT is BDNF. This factor can be produced locally by RGCs, astrocytes [69], and microglia [70], and it can play an important role in the survival of RGCs by protecting them from apoptosis. BDNF, by stimulating its high-affinity receptor, tropomyosin receptor kinase B (TrkB), causes the activation of prosurvival cell signaling pathways that induce neuroprotection in the retina [71,72]. The TrkB expression is relatively high in RGCs and Müller glia [73,74]. Stimulation of BDNF-TrkB signaling in glial cells has been shown to produce neuroprotective effects by supplying neurotrophic factors that stimulate neuroprotection [75]. The reduction in the retrograde transport of neurotrophic factors such as BDNF in RGCs, by increasing IOP, has been suggested as a critical factor for the degeneration of these cells in glaucoma [71,72]. The neuroprotective effect of BDNF was shown in an OHT model similar to that used in the present study but carried out in rats. BDNF produced neuroprotection of RGCs against OHT-induced retinal injury. Thus, approximately 25‒38% of Brn3a+ RGCs in the BDNF-treated groups were rescued after 12 or 15 days compared to the corresponding vehicle-treated groups [76]. In our study, we observed an upregulation of BDNF at all time points analyzed after OHT induction, finding immunolabeling in the macroglial cells. Disruption of retrograde transport by increased IOP may favor the release of BDNF in an attempt to maintain the survival of RGCs [71]. This was not observed, however, in the contralateral eye, where significant upregulation of BDNF did not occur because this factor would not be as necessary as in OHT eyes, due to the lower Brn3a+ RGCs decrease.

As in previous studies, we found a decrease in Brn3a+ RGCs in the OHT eyes in this mouse model of OHT. These studies were performed one week and later after OHT induction [42,43,44]. However, in the present work, we analyzed earlier times. The decrease in Brn3a+ RGCs in OHT was observed three days after laser induction and was more notable by day 5. The Brn3a+ RGCs’ decrease was initiated in the temporal region, in particular in its central zone, and spread to the central and, to a lesser extent, to the nasal retinal regions; in each region in the central and superior areas, the number of RGCs was lower than in the inferior one. The fact that the number of RGCs was lower at day 5 than at days 7 and 15 could be explained by a transitory loss of Brn3a expression in some RGCs that could have been recovered later; however, other cells could have been lost definitively. This transitory loss of expression of Brn3a+ could coincide with the greater activation of the microglial cells described in previous studies of our group, at days 3 and 5 in OHT eyes [21], and with the pro-inflammatory process we have described in the present study mainly at days 3 and 5 after OHT induction.

In contralateral eyes, we observed a minor decrease in Brn3a+ RGCs that only reached a slight significance at days 3 and 15. The decrease in Brn3a+ RGCs was initiated at day 3 in the temporal retinal region, with its superior and central zones being the only ones affected, and it was also significant at day 7, only in the temporal central retina, and at day 15 in the temporal central and temporal nasal retina. As occurred with OHT, a transient downregulation of Brn3a+ expression may occur in some RGCs. This Brn3a+ downregulation may coincide with increased microglial cell activation in the contralateral eye at days 3 and 5 after laser induction, although to a lesser degree than in the OHT eyes [21]. In addition, in the present study, we demonstrated an alteration in the levels of pro- and anti-inflammatory cytokines in contralateral eyes that could be caused by immune signals derived from the OHT eyes [6,8,9,20,44]; altogether, this could explain the downregulation in Brn3a+ expression detected in contralateral eyes. However, in two previous studies carried out with this experimental model [42,43], no significant differences were found in the number of RGCs in the contralateral eyes at days 7 and 15 after OHT induction. The slight differences observed between these studies and the present work could be due to the different quantification method used: our study used retinal sections, while previous works considered whole retinas.

## 4. Materials and Methods

### 4.1. Animals and Anesthetics

Albino male Swiss mice (12‒16 weeks of age and 40‒45 g in weight) from the University of Murcia breeding colony were used in this study. Animals were given free access to water and a standardized diet and were kept under a light intensity ranging from 9 to 25 lux and controlled temperature.

All procedures, OHT induction, and IOP measurement were performed under intraperitoneal general anesthesia with a mixture of xylazine (10 mg/kg; Xilagesic^®^, Laboratorios Carlier SA, Barcelona, Spain) and ketamine (75 mg/kg; Ketamidor^®^, Richter Pharma AG, Wels, Austria). To prevent possible desiccation and corneal infection after surgery, ocular topic tobramycin (Tobrex^®^; Novartis Farmaceutica SA, Barcelona, Spain) was applied on the corneal surface during the anesthesia recovering time. Animals were handled in such a way as to minimize discomfort and pain during the experiments. An intraperitoneal overdose of pentobarbital (Dolethal Vetoquinol^®^, Especialidades Veterinarias, Madrid, Spain) was used to kill the animals.

Experiments were performed in accordance with Spanish law and the Guidelines for Humane Endpoints for Animals Used in Biomedical Research. In addition, the study was approved by the Ethics Committee for Animal Research of Murcia University (Murcia, Spain) and the Animal Health Service of the Murcia Regional Ministry of Agriculture and Water (approval ID number: A13170110; approval date: 11 January 2017). All animal procedures were performed using the institutional guidelines, European Union regulations for the use of animals in research, and the Association for Research in Vision and Ophthalmology (ARVO) statement for the use of animals in ophthalmic and vision research.

### 4.2. Experimental Groups

Animals were divided into six groups: one naïve control group, not submitted to any procedure, and five OHT groups, in which animals were sacrificed at different time points after OHT laser induction (1, 3, 5, 7, and 15 days), and both the treated OHT eye and the untreated contralateral eyes were analyzed. In the present study, two separate sets of animals were employed. For the multiplexed immunoassay, 12 animals per experimental group were used, and five animals per group were used for the immunohistochemistry study.

### 4.3. Laser Induction and Measurement of IOP

A single session with a diode laser (spot size, 50‒100 μm; duration, 0.5 s; power, 0.3 W (Viridis Ophthalmic Photocoagulator-532 nm, Quantel Medical, Clermont-Ferrand, France) in the left eyes was used to induce OHT, as previously described [77,78]. The limbal and episcleral veins were photocoagulated by applying 55‒76 laser burns. The intraocular pressure (IOP) was measured in the treated eyes and in the contralateral untreated eyes with a rebound tonometer (Tono-Lab, Tiolat, OY, Helsinki, Finland) [79,80]. In the naïve group, IOP was registered before sacrifice. In the OHT groups, IOP was taken before laser induction and at different time points of the study (1, 2, 3, 5, 7, and 15 days). Possible variations in the IOP measures, due to circadian rhythms or spontaneous increases, were minimized by performing the measurement at the same time, 9 a.m. and immediately after anesthesia [81,82].

### 4.4. Multiplexed Immunoassay Study

#### 4.4.1. Protein Assay

The animals were sacrificed with a pentobarbital overdose, and then the retinas were dissected and snap frozen. Given the small amount of retinal tissue obtained from each mouse, three retinas were needed to obtain enough protein concentration to perform the assay. Thus, four samples (from three mice from the same experimental group) were employed in the present study. Retinal tissue was homogenized in a lysis buffer (MILLIPLEX MAP Lysis buffer for Multiplexing, Merck KGaA, Darmstadt, Germany), on ice, at a proportion of 1:3 (weight/volume) and then frozen overnight at −70 °C. The next day, samples were centrifuged at 12,000× *g* for 5 min at 4 °C. Supernatants were collected and centrifuged again with the same procedure. Final supernatants were transferred to an aliquot and total protein concentration was estimated by Bradford protein assay (Bio-Rad Dye Reagent Concentrate, Bio-Rad Laboratories, Irvine, CA, USA) and analyzed with Multiskan lector (ThermoFisher Technologies, Madrid, Spain). The total protein concentration obtained was sufficient to perform our immunoassay.

#### 4.4.2. Multiplexed Magnetic Bead Immunoassay

Cytokines and myokines were measured in duplicate using two multiplexed magnetic bead immunoassay kits (MILLIPLEX MAP Mouse Cytokine/ Chemokine Magnetic Bead Panel; MILLIPLEX MAP Myokine Magnetic Bead Panel, Merck KGaA, Darmstadt, Germany). This method of analysis is based on the Luminex^©^ technology. In summary, retinal tissue samples (25 µL) and magnetic beads (25 µL) conjugated to the specific antibody for different cytokines/myokines (IFN-γ, IL-1β, IL-4, IL-6, IL-10, IL-17, TNF-α, VEGF, BDNF, and fractalkine) were incubated with shaking overnight at 4 °C. After that, wells were washed three times using a wash buffer and a biotinylated antibody was added for detection. We incubated those for 1 h at room temperature. After incubation, beads were incubated with streptavidin-PE (Phycoerythin), a reporter molecule that completes the reaction on the surface of each microsphere for 30 min, at room temperature. Samples were washed three more times and the detection compound included in each immunoassay kit was added. Samples incubated with conjugated beads were analyzed using the Bio-Plex suspension array system 200 and mean fluorescence intensity was analyzed using Bio-Plex Manager Software 4.1 (Bio-Rad Laboratories, Irvine, CA, USA).

### 4.5. Immunostaining

Animals were deeply anesthetized, as mentioned previously, and transcardially perfused with 0.9% saline solution followed by 4% paraformaldehyde (PFA 4%) in a 0.1 M phosphate buffer. Eyes were removed and post-fixed for 24 h, at 4 °C, in the same fixative solution. Twenty-four hours later, corneas and lenses were removed and optical cups with the retina were kept in the same solution overnight at 4 °C. The day after, they were washed three times, 30 min each, in phosphate buffer saline (PBS), pH 7.2, and cryoprotected in 11% sucrose in PBS at 4 °C, for 24 h; samples were then transferred to PBS containing 33% sucrose and conserved at 4 °C for 48 h. Finally, the samples were embedded in a tissue-freezing medium (Tissue-Tek^®^ O.C.T.™ Compound, Sakura Finetek Spain, Barcelona, Spain), preserving eye anatomical references in order to assure the spatial orientation of the retinas, and kept at −30 °C until use.

For immunostaining techniques, optical cups with retinas were frozen sectioned using a Leica cryostat CM-3050 (Leica Biosystems, Heidelberger, Germany) in 16 μm-thick serial sagittal sections from the nasal to temporal retina (Figure 1). Tissue sections were collected onto gelatin-coated slides (two sections per slide), air-dried, and stored at −30 °C until use.

Immunohistochemical techniques were used to evaluate which cells of the retina expressed the different cytokines and factors analyzed in the multiplex assay. We selected those eyes (OHT and/or contralateral) and time points where their expression was greatest, showing the most notable changes in our glaucoma model; namely, IL-1β, IL-6, IL-17, IFN-γ, BDNF, VEGF, CX3CL1, TNF-α, IL-4, and IL-10. Colocalization of these cytokines with microglial cells, macroglia (astrocytes and Müller cells), and retinal ganglion cells (soma or axonal neurofilaments), identified with Iba-1 (red fluorochrome-conjugated), GFAP, and Brn3a and NF-200 (see details in Table 2) antibodies, respectively, was performed by double-labeling fluorescent immunohistochemistry.

Slides were allowed to dry at room temperature for 60 min, in order to increase the adhesion of the slices to the slides. All washes were conducted in PBS, pH 7.2, containing 0.1% Triton X-100, which constituted the washing buffer (WB), while incubations were performed in PBS, pH 7.2, containing 0.1% Triton X-100 and 10% R.T.U Animal-Free Blocker and Diluent, (SP-5035; Vector Laboratories, Inc., Burlingame, CA, USA), which constituted the immunohistochemistry buffer (IB). After three washes in WB, sections were incubated overnight at 4 °C with the primary antibodies (see Table 1), then rinsed three times in WB, and incubated for 2 h at room temperature with the secondary antibodies, except for Iba-1 immunostaining (not needed). Details and dilutions of all the primary and secondary antibodies used are presented in Table 2.

After incubations, sections were washed three more times with WB, then coverslipped with a Vectashield Vibrance Antifade^®^ mounting medium with DAPI (Ref. H-1800; Vector Laboratories, Burlingame, CA, USA).

Immunostaining batches contained slides of the nasal, central, and temporal retina of every animal from the selected experimental group (*n* = 5 per experimental group), as well as an internal control (omitting the primary antibody), to check the specificity of the immunoreaction and rule out unspecific binding. Three different batches were run for each primary antibody.

Immunostained slides were observed under a fluorescence microscope Zeiss Axio Imager M.2 (Carl Zeiss AG, Oberkochen, Germany) associated with the Apotome-2 module (Carl Zeiss AG, Oberkochen, Germany) and high-resolution camera Axio Cam 503 Mono (Carl Zeiss AG, Oberkochen, Germany). The microscope was equipped with a Zeiss 10 filter set for Alexa Fluor 488, a Zeiss 64 filter set for Alexa Fluor 594, and a 49 filter set for Alexa Fluor 405. Images taken were analyzed using ZEN2 software (Carl Zeiss AG, Oberkochen, Germany). All lighting conditions and magnifications were kept constant during the capture process. Figures were prepared using Adobe Photoshop CS4 Extended 10.0 (Adobe Systems, San Jose, CA, USA).

### 4.6. Retinal Ganglion Cells’ (RGCs) Quantification

**Figure 14:**
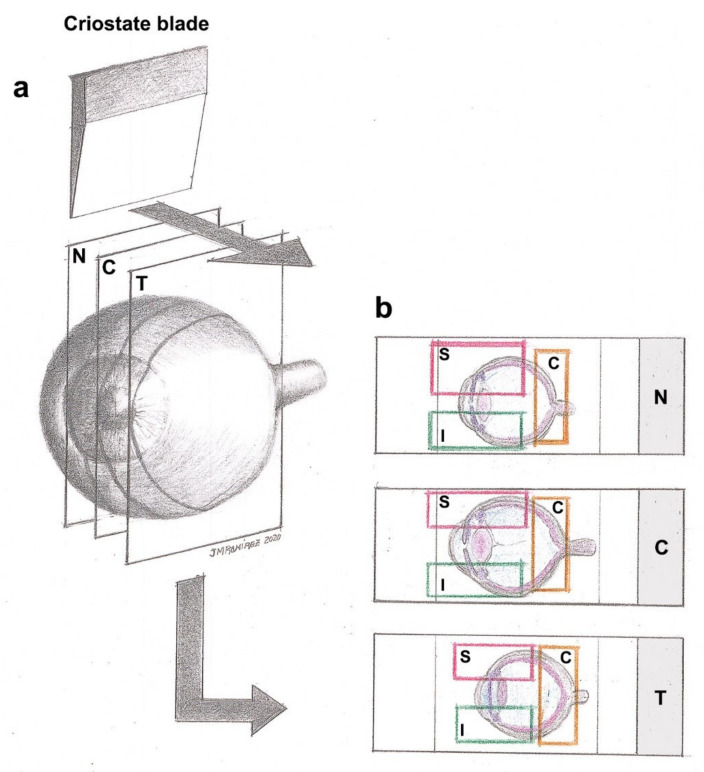
Diagram of the retina showing the nine spatial areas analyzed. (**a**) Each optical cup with the retina was frozen sectioned in 16 μm-thick serial sagittal sections from nasal to temporal (nasal, central, and temporal) retina. (**b**) Tissue sections were collected onto gelatin-coated slides. Images were obtained at 20× magnification from each region (nasal, central, and temporal) and zone of the retina (superior, central, and inferior), and equivalent areas of the retina were consistently selected in all slices (rectangle in (**b**)). Immunostaining batches contained sections from the nine spatial areas indicated: nasal‒superior, nasal‒central, and nasal‒inferior; central‒superior, central‒central, and central‒inferior; and temporal‒superior, temporal‒central, and temporal‒inferior. Abbreviations: N (nasal); T (temporal); C (central); S (superior); I (inferior).

We quantified the number of Brn3a-positive (Brn3a+) RGCs using a double-blind procedure: counts were performed on coded slides, with unbiased evaluation; one slide per retinal region (nasal, central, and temporal) and animal was randomly selected, and two tissue sections per slide were analyzed. Quantification of Brn3a+ RGCs was performed in OHT eyes (*n* = 5), contralateral eyes (*n* = 5), and naïve eyes (*n* = 5) on high-resolution digital microphotographs, which were captured at the 20x magnification objective. The number of Brn3a+ RGCs was estimated by counting the number of immunoreactive Brn3a+ cells in the RGCs layer (Dogiel cells were not included in the quantification) of two consecutive microphotographs (representing a retinal area of 0.3004 mm^2^) from each region (nasal, central, and temporal) of the retina and within these regions in three zones (superior, central, and inferior). Equivalent areas of the retina were consistently selected in all slices. Therefore, we counted all the immunoreactive Brn3a+ cells that came into focus in nine spatial areas of the retina: nasal‒superior, nasal‒central, and nasal‒inferior; central‒superior, central‒central, and central‒inferior; and temporal‒superior, temporal‒central, and temporal‒inferior (Figure 14).

### 4.7. Statistical Analysis

The IOP measures, the Luminex values for different cytokines/myokines, and RGCs’ quantification were analyzed using SPSS version 25 (IBM, Armonk, NY, USA) and reported as the mean (±standard deviation, SD). The significant differences among naïve, contralateral, and OHT eyes were determined using a two-way analysis of variance (ANOVA) with Bonferroni test correction. A *p*-value less than 0.05 was considered statistically significant.

## 5. Conclusions

We can conclude that increased IOP causes changes in the levels of pro-inflammatory and anti-inflammatory cytokines, BDNF, VEGF, and fractalkine, in a mouse experimental model of unilateral laser-induced OHT, both in OHT eyes and in contralateral normotensive eyes, associated with the neurodegenerative process. At the earliest time points (days 1, 3, and 5 after OHT laser induction), the expression of pro-inflammatory cytokines would be compensated for by the expression of anti-inflammatory cytokines, in an attempt to control the damage to the RGCs. However, more prolonged exposure to pro-inflammatory factors such as IFN-γ could lead to the death of RGCs from three days after OHT laser induction, as we observed in this study. The main changes in the expression of cytokines and other factors occurring at days 1, 3, and 5 after laser induction may be related to the activation of microglial cells. In previous works, we observed that at these time points (days 1, 3, and 5), the main signs of microglial activation are seen in the eyes of OHT, which would support the results of this study. Further, in normotensive contralateral eyes (where there is a modest decrease in the number of Brn3a+ RGCs), changes in the levels of cytokines and other molecules occur, although they are slighter than those observed in OHT eyes, which would coincide with the signs of microglial activation observed in contralateral eyes in previous studies. Therefore, with this study, we can confirm the participation of the immune system in glaucomatous neurodegeneration.

## Figures and Tables

**Table 1:** Multiplexed immunoassay and immunohistochemical results.

Cytokine	Multiplex	Time point	*p*-Value	Co-Expression Cell Type
IL-1β	Downregulated OHT eyes	1 day 5 days 7 days	*p <* 0.01 *p <* 0.05 *p <* 0.01	Microglia and Macroglia (astrocytes and Müller cells)
	Upregulated Contralateral eyes	1 day 3 days	*p <* 0.05 *p <* 0.01	
	Downregulated Contralateral eyes	7 days	*p <* 0.01	
IL-6	Upregulated OHT eyes	1 and 3 days 5 days	*p <* 0.01 *p <* 0.05	Microglia
	Upregulated Contralateral eyes	1, 5, and 7 days	*p <* 0.05	
	Downregulated Contralateral eyes	15 days	*p <* 0.05	
INF-γ	Upregulated OHT eyes	3 days 5 days 15 days	*p <* 0.01 *p <* 0.05 *p <* 0.01	Microglia
	No difference Contralateral eyes			
IL-17	Downregulated OHT eyes	All time points	*p <* 0.01	Microglia
	Upregulated Contralateral eyes	15 days	*p <* 0.01	
TNF-α	No detectable concentration			Microglia and Astrocytes
IL-4	Upregulated OHT eyes	1, 3, 5, and 7 days	*p <* 0.01	Microglia
	Upregulated Contralateral eyes	7 and 15 days	*p <* 0.01	
IL-10	Upregulated OHT eyes	3 days 5 days	*p <* 0.05 *p <* 0.01	Axons of retinal ganglion cells
	Downregulated OHT eyes	7 days	*p <* 0.05	
	Upregulated Contralateral eyes	3 days 7 days	*p <* 0.01 *p <* 0.05	
BDNF	Upregulated OHT eyes	1, 3, 7, and 15 days	*p <* 0.01	Macroglia (astrocytes and Müller cells)
	Downregulated Contralateral eyes	1 day 3 and 15 days	*p <* 0.01 *p <* 0.05	
VEGF	Upregulated OHT eyes	1 day	*p <* 0.01	Macroglia (astrocytes and Müller cells)
	Downregulated OHT eyes	7 and 15 days	*p <* 0.01	
	Downregulated Contralateral eyes	1, 7, and 15 days	*p <* 0.01	
Fractalkine	Upregulated OHT eyes	1 day	*p <* 0.01	Microglia
	Downregulated OHT eyes	3, 5, and 7 days 15 days	*p <* 0.01 *p <* 0.05	
	Downregulated Contralateral eyes	1, 3, and 5 days	*p <* 0.01	

Results of different cytokine concentrations in the OHT and contralateral eyes in comparison with naïve eyes at different time points after laser-induced ocular hypertension, as well as the co-expression of these same cytokines with microglia, macroglia (astrocytes and Müller cells), and retinal ganglion cell axons.

**Table 2:** Antibodies employed for the immunostaining analysis.

Color	Primary Antibody	Conc.	Secondary Antibody	Conc.
GREEN	Rabbit polyclonal anti IL1β (ref. ab9722, Abcam plc) [83]	1:250	Goat anti rabbit Alexa Fluor 488 (ref. ab150077, Abcam plc)	1:150
	Rabbit polyclonal anti IL6 (ref. ab208113, Abcam plc) [84]	1:200		
	Rabbit polyclonal anti IL17 (ref. ab79056, Abcam plc) [85]	1:300		
	Rabbit polyclonal anti IFNγ (ref. ab9657, Abcam plc) [86]	1:300		
	Rabbit monoclonal anti BDNF (ref. ab213323, Abcam plc) [83]	1:250		
	Rabbit monoclonal anti VEGF receptor 1 (ref. ab32152, Abcam plc) [87]	1:200		
	Rabbit polyclonal anti CX3CL1 (ref. ab25088, Abcam plc) [88]	1:500		
	Rabbit polyclonal anti TNFα (ref. ab9739, Abcam plc) [89]	1:300		
	Rat monoclonal anti IL4 (ref. ab11524, Abcam plc) [90]	1:250	Goat anti rat Alexa Fluor 488 (ref. ab150165, Abcam plc)	1:150
	Rat monoclonal anti IL10 (ref. ab189392, Abcam plc) [91]	1:200		
RED	Rabbit polyclonal anti Iba-1 Red Fluorochrome 635 conjugated (ref. 5100756, Wako Chemicals GmbH) [92]	1:200		
	Mouse monoclonal anti Brn-3a (ref. MAB1585, Sigma-Aldrich) [93]	1:600	Goat anti mouse IgG1 Alexa Fluor 594 (ref. A21125, Invitrogen)	1:1000
	Chicken polyclonal anti GFAP (ref. AB5541, Sigma-Aldrich) [94]	1:200	Goat anti chicken IgY (H + L) Alexa Fluor 594 (ref. A-11042, Invitrogen)	1:300
	Rabbit polyclonal anti NF-200 (ref. N4142, Sigma-Aldrich) [95]	1:150	Donkey anti rabbit IgG1 Alexa Fluor 594 (ref. A21207, Invitrogen)	1:800

Details of the commercial antibodies employed are indicated, including the concentration at which they were used. The antibodies employed for the determination of cytokines include: interleukin 1 beta (IL-1β), interleukin 6 (IL-6), interleukin 17 (IL-17), interferon gamma (IFN-γ), brain-derived neurotrophic factor (BDNF), vascular endothelial growth factor (VEGF), fractalkine (CX3CL1), tumor necrosis factor alpha (TNF-α), interleukin 4 (IL-4), and interleukin 10 (IL-10); as well as those employed to identify microglial cells, Iba-1; retinal ganglion cells, Brn3a (soma) and NF-200 KDa (axonal neurofilaments); and macroglia, GFAP. The color (green/red) indicates how the immunostaining is labeled.

## Data Availability

The data supporting the findings of this study are available from the corresponding author upon request.

## References

[1] Quigley H., Broman A.T. (2006). The number of people with glaucoma worldwide in 2010 and 2020. Br. J. Ophthalmol..

[2] Qu J., Wang D., Grosskreutz C.L. (2010). Mechanisms of retinal ganglion cell injury and defense in glaucoma. Exp. Eye Res..

[3] Baltmr A., Duggan J., Nizari S., Salt T.E., Cordeiro M.F. (2010). Neuroprotection in glaucoma—Is there a future role?. Exp. Eye Res..

[4] Tezel G., Ben-Hur T., Gibson G.E., Stevens B., Streit W.J., Wekerle H., Bhattacharya S.K., Borras T., Burgoyne C.F., Caspi R.R. (2009). The role of glia, mitochondria, and the immune system in glaucoma. Investig. Ophthalmol. Vis. Sci..

[5] Davis B.M., Salinas-Navarro M., Cordeiro M.F., Moons L., Groef L. (2017). De Characterizing microglia activation: A spatial statistics approach to maximize information extraction. Sci. Rep..

[6] Gallego B.I., Salazar J.J., de Hoz R., Rojas B., Ramírez A.I., Salinas-Navarro M., Ortín-Martínez A., Valiente-Soriano F.J., Avilés-Trigueros M., Villegas-Perez M.P. (2012). IOP induces upregulation of GFAP and MHC-II and microglia reactivity in mice retina contralateral to experimental glaucoma. J. Neuroinflamm..

[7] Ramirez A.I., de Hoz R., Salobrar-García E., Salazar J.J., Rojas B., Ajoy D., López-Cuenca I., Rojas P., Triviño A., Ramírez J.M. (2017). The role of microglia in retinal neurodegeneration: Alzheimer’s disease, Parkinson, and glaucoma. Front. Aging Neurosci..

[8] De Hoz R., Gallego B.I., Ramírez A.I., Rojas B., Salazar J.J., Valiente-Soriano F.J., Avilés-Trigueros M., Villegas-Perez M.P., Vidal-Sanz M., Triviño A. (2013). Rod-like microglia are restricted to eyes with laser-induced ocular hypertension but absent from the microglial changes in the contralateral untreated eye. PLoS ONE.

[9] Rojas B., Gallego B.I., Ramírez A.I., Salazar J.J., de Hoz R., Valiente-Soriano F.J., Avilés-Trigueros M., Villegas-Perez M.P., Vidal-Sanz M., Triviño A. (2014). Microglia in mouse retina contralateral to experimental glaucoma exhibit multiple signs of activation in all retinal layers. J. Neuroinflamm..

[10] Ramírez A.I., Rojas B., de Hoz R., Salazar J.J., Gallego B., Triviño A., Ramírez J.M. (2015). Microglia, Inflammation, and Glaucoma. Glaucoma.

[11] Karlstetter M., Scholz R., Rutar M., Wong W.T., Provis J.M., Langmann T. (2015). Retinal microglia: Just bystander or target for therapy?. Prog. Retin. Eye Res..

[12] Kettenmann H., Hanisch U.K., Noda M., Verkhratsky A. (2011). Physiology of microglia. Physiol. Rev..

[13] Borkenstein A., Faschinger C., Maier R., Weger M., Theisl A., Demel U., Graninger W., Irene H., Mossböck G. (2013). Measurement of tumor necrosis factor-alpha, interleukin-6, Fas ligand, interleukin-1α, and interleukin-1β in the aqueous humor of patients with open angle glaucoma using multiplex bead analysis. Mol. Vis..

[14] Almasieh M., Wilson A.M., Morquette B., Cueva Vargas J.L., Di Polo A. (2012). The molecular basis of retinal ganglion cell death in glaucoma. Prog. Retin. Eye Res..

[15] Chua J., Vania M., Cheung C.M.G., Ang M., Chee S.P., Yang H., Li J., Wong T.T. (2012). Expression profile of inflammatory cytokines in aqueous from glaucomatous eyes. Mol. Vis..

[16] Huang W., Fileta J., Rawe I., Qu J., Grosskreutz C.L. (2010). Calpain activation in experimental glaucoma. Investig. Ophthalmol. Vis. Sci..

[17] Hu D.N., Ritch R., Liebmann J., Liu Y., Cheng B., Hu M.S. (2002). Vascular endothelial growth factor is increased in aqueous humor of glaucomatous eyes. J. Glaucoma.

[18] Liu X., Huang P., Wang J., Yang Z., Huang S., Luo X., Qi J., Shen X., Zhong Y. (2016). The effect of A2A receptor antagonist on microglial activation in experimental glaucoma. Investig. Ophthalmol. Vis. Sci..

[19] Russo R., Varano G.P., Adornetto A., Nucci C., Corasaniti M.T., Bagetta G., Morrone L.A. (2016). Retinal ganglion cell death in glaucoma: Exploring the role of neuroinflammation. Eur. J. Pharmacol..

[20] de Hoz R., Ramírez A.I., González-Martín R., Ajoy D., Rojas B., Salobrar-García E., Valiente-Soriano F.J., Avilés-Trigueros M., Villegas-Pérez M.P., Vidal-Sanz M. (2018). Bilateral early activation of retinal microglial cells in a mouse model of unilateral laser-induced experimental ocular hypertension. Exp. Eye Res..

[21] Ramírez A.I., de Hoz R., Fernández-Albarral J.A., Salobrar-García E., Rojas B., Valiente-Soriano F.J., Avilés-Trigueros M., Villegas-Pérez M.P., Vidal-Sanz M., Triviño A. (2020). Time course of bilateral microglial activation in a mouse model of laser-induced glaucoma. Sci. Rep..

[22] Haynes S.E., Hollopeter G., Yang G., Kurpius D., Dailey M.E., Gan W.-B., Julius D. (2006). The P2Y12 receptor regulates microglial activation by extracellular nucleotides. Nat. Neurosci..

[23] Varnum M.M., Ikezu T. (2012). The classification of microglial activation phenotypes on neurodegeneration and regeneration in alzheimer’s disease brain. Arch. Immunol. Ther. Exp..

[24] González H., Elgueta D., Montoya A., Pacheco R. (2014). Neuroimmune regulation of microglial activity involved in neuroinflammation and neurodegenerative diseases. J. Neuroimmunol..

[25] Jones E.V., Bouvier D.S. (2014). Astrocyte-secreted matricellular proteins in CNS remodelling during development and disease. Neural Plast..

[26] Suh H.S., Zhao M.L., Derico L., Choi N., Lee S.C. (2013). Insulin-like growth factor 1 and 2 (IGF1, IGF2) expression in human microglia: Differential regulation by inflammatory mediators. J. Neuroinflamm..

[27] Tang Y., Le W. (2016). Differential Roles of M1 and M2 Microglia in Neurodegenerative Diseases. Mol. Neurobiol..

[28] Langmann T. (2007). Microglia activation in retinal degeneration. J. Leukoc. Biol..

[29] Nakagawa Y., Chiba K. (2014). Role of microglial M1/M2 polarization in relapse and remission of psychiatric disorders and diseases. Pharmaceuticals.

[30] Ivashkiv L.B. (2018). IFNγ: Signalling, epigenetics and roles in immunity, metabolism, disease and cancer immunotherapy. Nat. Rev. Immunol..

[31] Kaur C., Rathnasamy G., Ling E.A. (2013). Roles of activated microglia in hypoxia induced neuroinflammation in the developing brain and the retina. J. Neuroimmune Pharmacol..

[32] Crish S.D., Sappington R.M., Inman D.M., Horner P.J., Calkins D.J. (2010). Distal axonopathy with structural persistence in glaucomatous neurodegeneration. Proc. Natl. Acad. Sci. USA.

[33] Madeira M.H., Ortin-Martinez A., Nadal-Nícolas F., Ambrósio A.F., Vidal-Sanz M., Agudo-Barriuso M., Santiago A.R. (2016). Caffeine administration prevents retinal neuroinflammation and loss of retinal ganglion cells in an animal model of glaucoma. Sci. Rep..

[34] Chidlow G., Wood J.P.M., Ebneter A., Casson R.J. (2012). Interleukin-6 is an efficacious marker of axonal transport disruption during experimental glaucoma and stimulates neuritogenesis in cultured retinal ganglion cells. Neurobiol. Dis..

[35] Yuan L., Neufeld A.H. (2000). Tumor necrosis factor-α: A potentially neurodestructive cytokine produced by glia in the human glaucomatous optic nerve head. Glia.

[36] Koeberle P.D., Gauldie J., Ball A.K. (2004). Effects of adenoviral-mediated gene transfer of interleukin-10, interleukin-4, and transforming growth factor-β on the survival of axotomized retinal ganglion cells. Neuroscience.

[37] Hart P.H., Cooper R.L., Finlay-Jones J.J. (1991). IL-4 suppresses IL-1 beta, TNF-alpha and PGE2 production by human peritoneal macrophages. Immunology.

[38] Walker S.O., Kolostyak D.Y., Wu F.N., Ros-Cisneros A.A. (2001). IL-10 receptor localization on naive retinal ganglion cells. Investig. Ophthalmol. Vis. Sci..

[39] Wilson G.N., Inman D.M., Denger-Crish C.M., Smith M.A., Crish S.D. (2015). Early pro-inflammatory cytokine elevations in the DBA/2J mouse model of glaucoma. J. Neuroinflamm..

[40] Johnson E.C., Doser T.A., Cepurna W.O., Dyck J.A., Jia L., Guo Y., Lambert W.S., Morrison J.C. (2011). Cell proliferation and interleukin-6-type cytokine signaling are implicated by gene expression responses in early optic nerve head injury in rat glaucoma. Investig. Ophthalmol. Vis. Sci..

[41] Sappington R.M., Chan M., Calkins D.J. (2006). Interleukin-6 protects retinal ganglion cells from pressure-induced death. Investig. Ophthalmol. Vis. Sci..

[42] Salinas-Navarro M., Alarcón-Martínez L., Valiente-Soriano F.J., Ortín-Martínez A., Jiménez-López M., Avilés-Trigueros M., Villegas-Pérez M.P., de la Villa P., Vidal-Sanz M. (2009). Functional and morphological effects of laser-induced ocular hypertension in retinas of adult albino Swiss mice. Mol. Vis..

[43] Fernández-Albarral J.A., Ramírez A.I., de Hoz R., López-Villarín N., Salobrar-García E., López-Cuenca I., Licastro E., Inarejos-García A.M., Almodóvar P., Pinazo-Durán M.D. (2019). Neuroprotective and anti-inflammatory effects of a hydrophilic saffron extract in a model of glaucoma. Int. J. Mol. Sci..

[44] Vidal-Sanz M., Salinas-Navarro M., Nadal-Nicolás F.M., Alarcón-Martínez L., Valiente-Soriano F.J., de Imperial J.M., Avilés-Trigueros M., Agudo-Barriuso M., Villegas-Pérez M.P. (2012). Understanding glaucomatous damage: Anatomical and functional data from ocular hypertensive rodent retinas. Prog. Retin. Eye Res..

[45] Fernández-Albarral J.A., Salobrar-García E., Martínez-Páramo R., Ramírez A.I., de Hoz R., Ramírez J.M., Salazar J.J. (2019). Retinal glial changes in Alzheimer’s disease—A review. J. Optom..

[46] Echevarria F.D., Formichella C.R., Sappington R.M. (2017). Interleukin-6 deficiency attenuates retinal ganglion cell axonopathy and glaucoma-related vision loss. Front. Neurosci..

[47] Gaffen S.L. (2009). Structure and signalling in the IL-17 receptor family. Nat. Rev. Immunol..

[48] Tzartos J.S., Friese M.A., Craner M.J., Palace J., Newcombe J., Esiri M.M., Fugger L. (2008). Interleukin-17 production in central nervous system-infiltrating T cells and glial cells is associated with active disease in multiple sclerosis. Am. J. Pathol..

[49] Chien Y.H., Zeng X., Prinz I. (2013). The natural and the inducible: Interleukin (IL)-17-producing γδ T cells. Trends Immunol..

[50] Kawanokuchi J., Shimizu K., Nitta A., Yamada K., Mizuno T., Takeuchi H., Suzumura A. (2008). Production and functions of IL-17 in microglia. J. Neuroimmunol..

[51] Gravallese E.M., Monach P.A., Hochberg M.C., Silman A.J., Smolen J.S., Weinblatt M.E., Weisman M.H. (2015). The Rheumatoid Joint: Synovitis and Tissue Destruction. Rheumatology: Sixth Edition.

[52] Chabaud M., Fossiez F., Taupin J.L., Miossec P. (1998). Enhancing effect of IL-17 on IL-1-induced IL-6 and leukemia inhibitory factor production by rheumatoid arthritis synoviocytes and its regulation by Th2 cytokines. J. Immunol..

[53] Hu M.H., Zheng Q.F., Jia X.Z., Li Y., Dong Y.C., Wang C.Y., Lin Q.Y., Zhang F.Y., Zhao R.B., Xu H.W. (2014). Neuroprotection effect of interleukin (IL)-17 secreted by reactive astrocytes is emerged from a high-level IL-17-containing environment during acute neuroinflammation. Clin. Exp. Immunol..

[54] Ke Y., Liu K., Huang G.-Q., Cui Y., Kaplan H.J., Shao H., Sun D. (2009). Anti-inflammatory role of IL-17 in experimental autoimmune uveitis. J. Immunol..

[55] Silverman M.D., Zamora D.O., Pan Y., Texeira P.V., Baek S.H., Planck S.R., Rosenbaum J.T. (2003). Constitutive and inflammatory mediator-regulated fractalkine expression in human ocular tissues and cultured cells. Investig. Ophthalmol. Vis. Sci..

[56] Liang K.J., Lee J.E., Wang Y.D., Ma W., Fontainhas A.M., Fariss R.N., Wong W.T. (2009). Regulation of dynamic behavior of retinal microglia by CX3CR1 signaling. Investig. Ophthalmol. Vis. Sci..

[57] Sokolowski J.D., Chabanon-Hicks C.N., Han C.Z., Heffron D.S., Mandell J.W. (2014). Fractalkine is a “find-me” signal released by neurons undergoing ethanol-induced apoptosis. Front. Cell. Neurosci..

[58] Cardona A.E., Pioro E.P., Sasse M.E., Kostenko V., Cardona S.M., Dijkstra I.M., Huang D.R., Kidd G., Dombrowski S., Dutta R. (2006). Control of microglial neurotoxicity by the fractalkine receptor. Nat. Neurosci..

[59] Breen K.T., Anderson S.R., Steele M.R., Calkins D.J., Bosco A., Vetter M.L. (2016). Loss of fractalkine signaling exacerbates axon transport dysfunction in a chronic model of glaucoma. Front. Neurosci..

[60] Landsman L., Liat B.O., Zernecke A., Kim K.W., Krauthgamer R., Shagdarsuren E., Lira S.A., Weissman I.L., Weber C., Jung S. (2009). CX3CR1 is required for monocyte homeostasis and atherogenesis by promoting cell survival. Blood.

[61] Sennlaub F., Auvynet C., Calippe B., Lavalette S., Poupel L., Hu S.J., Dominguez E., Camelo S., Levy O., Guyon E. (2013). CCR2+ monocytes infiltrate atrophic lesions in age-related macular disease and mediate photoreceptor degeneration in experimental subretinal inflammation in Cx3cr1 deficient mice. EMBO Mol. Med..

[62] Barleon B., Sozzani S., Zhou D., Weich H.A., Mantovani A., Marmé D. (1996). Migration of human monocytes in response to vascular endothelial growth factor (VEGF) is mediated via the VEGF receptor flt-1. Blood.

[63] Aiello L.P., Northrup J.M., Keyt B.A., Takagi H., Iwamoto M.A. (1995). Hypoxic Regulation of Vascular Endothelial Growth Factor in Retinal Cells. Arch. Ophthalmol..

[64] Miller J.W., Adamis A.P., Aiello L.P. (1997). Vascular Endothelial Growth Factor in Ocular Neovascularization and Proliferative Diabetic Retinopathy. Diabetes Metab. Rev..

[65] Pierce I.A., Avery R.L., Foley E.D., Aiello L.P., Smith L.E.H. (1995). Vascular endothelial growth factor/vascular permeability factor expression in a mouse model of retinal neovascularization. Proc. Natl. Acad. Sci. USA.

[66] Stone J., Itin A., Alon T., Pe’er J., Gnessin H., Chan-Ling T., Keshet’ E. (1995). Development of Retinal Vasculature Is Mediated by Hypoxia-Induced Vascular Endothelial Growth Factor (VEGF) Expression by Neuroglia. J. Neurosci..

[67] Stone J., Chan-Ling T., Pe’er J., Itin A., Gnessin H., Keshet E. (1996). Roles of vascular endothelial growth factor and astrocyte degeneration in the genesis of retinopathy of prematurity. Investig. Ophthalmol. Vis. Sci..

[68] Wang J., Xu E., Elliott M.H., Zhu M., Le Y.Z. (2010). Müller cell-derived VEGF is essential for diabetes-induced retinal inflammation and vascular leakage. Diabetes.

[69] Herzog K.H., Von Bartheld C.S. (1998). Contributions of the optic tectum and the retina as sources of brain- derived neurotrophic factor for retinal ganglion cells in the chick embryo. J. Neurosci..

[70] Rashid K., Akhtar-Schaefer I., Langmann T. (2019). Microglia in Retinal Degeneration. Front. Immunol..

[71] Quigley H.A., McKinnon S.J., Zack D.J., Pease M.E., Kerrigan-Baumrind L.A., Kerrigan D.F., Mitchell R.S. (2000). Retrograde axonal transport of BDNF in retinal ganglion cells is blocked by acute IOP elevation in rats. Investig. Ophthalmol. Vis. Sci..

[72] Gupta V., You Y., Li J., Gupta V., Golzan M., Klistorner A., van den Buuse M., Graham S. (2014). BDNF impairment is associated with age-related changes in the inner retina and exacerbates experimental glaucoma. Biochim. Biophys. Acta Mol. Basis Dis..

[73] Wahlin K.J., Campochiaro P.A., Zack D.J., Adler R. (2000). Neurotrophic factors cause activation of intracellular signaling pathways in Müller cells and other cells of the inner retina, but not photoreceptors. Investig. Ophthalmol. Vis. Sci..

[74] Grishanin R.N., Yang H., Liu X., Donohue-Rolfe K., Nune G.C., Zang K., Xu B., Duncan J.L., LaVail M.M., Copenhagen D.R. (2008). Retinal TrkB receptors regulate neural development in the inner, but not outer, retina. Mol. Cell. Neurosci..

[75] Kimura A., Namekata K., Guo X., Harada C., Harada T. (2016). Neuroprotection, Growth Factors and BDNF-TrkB Signalling in Retinal Degeneration. Int. J. Mol. Sci..

[76] Valiente-Soriano F.J., Nadal-Nicolás F.M., Salinas-Navarro M., Jiménez-López M., Bernal-Garro J.M., Villegas-Pérez M.P., Agudo-Barriuso M., Vidal-Sanz M. (2015). BDNF rescues RGCs but not intrinsically photosensitive RGCs in ocular hypertensive albino rat retinas. Investig. Ophthalmol. Vis. Sci..

[77] Cuenca N., Pinilla I., Fernández-Sánchez L., Salinas-Navarro M., Alarcón-Martínez L., Avilés-Trigueros M., de la Villa P., Miralles de Imperial J., Villegas-Pérez M.P., Vidal-Sanz M. (2010). Changes in the inner and outer retinal layers after acute increase of the intraocular pressure in adult albino Swiss mice. Exp. Eye Res..

[78] Salinas-Navarro M., Alarcón-Martínez L., Valiente-Soriano F.J., Jiménez-López M., Mayor-Torroglosa S., Avilés-Trigueros M., Villegas-Pérez M.P., Vidal-Sanz M. (2010). Ocular hypertension impairs optic nerve axonal transport leading to progressive retinal ganglion cell degeneration. Exp. Eye Res..

[79] Naskar R., Wissing M., Thanos S. (2002). Detection of early neuron degeneration and accompanying microglial responses in the retina of a rat model of glaucoma. Investig. Ophthalmol. Vis. Sci..

[80] Quigley H.A., Hohman R.M. (1983). Laser energy levels for trabecular meshwork damage in the primate eye. Investig. Ophthalmol. Vis. Sci..

[81] Neufeld A.H. (1999). Microglia in the optic nerve head and the region of parapapillary chorioretinal atrophy in glaucoma. Arch. Ophthalmol..

[82] Yuan L., Neufeld A.H. (2001). Activated microglia in the human glaucomatous optic nerve head. J. Neurosci. Res..

[83] Ding H., Chen J., Su M., Lin Z., Zhan H., Yang F., Li W., Xie J., Huang Y., Liu X. (2020). BDNF promotes activation of astrocytes and microglia contributing to neuroinflammation and mechanical allodynia in cyclophosphamide-induced cystitis. J. Neuroinflamm..

[84] Guo X., Wang X., Dong J., Lv W., Zhao S., Jin L., Guo J., Wang M., Cai C., Sun J. (2020). Effects of Ginkgo biloba on Early Decompression after Spinal Cord Injury. Evid. Based Complement. Altern. Med..

[85] Sun L., Zhang H., Wang W., Chen Z., Wang S., Li J., Li G., Gao C., Sun X. (2020). Astragaloside IV Exerts Cognitive Benefits and Promotes Hippocampal Neurogenesis in Stroke Mice by Downregulating Interleukin-17 Expression via Wnt Pathway. Front. Pharmacol..

[86] Johnson S., Duncan J., Hussain S.A., Chen G., Luo J., Mclaurin C., May W., Rajkowska G., Ou X.M., Stockmeier C.A. (2015). The IFNγ-PKR pathway in the prefrontal cortex reactions to chronic excessive alcohol use. Alcohol. Clin. Exp. Res..

[87] Vaquié A., Sauvain A., Duman M., Nocera G., Egger B., Meyenhofer F., Falquet L., Bartesaghi L., Chrast R., Lamy C.M. (2019). Injured Axons Instruct Schwann Cells to Build Constricting Actin Spheres to Accelerate Axonal Disintegration. Cell Rep..

[88] Chen G., Zhou Z., Sha W., Wang L., Yan F., Yang X., Qin X., Wu M., Li D., Tian S. (2020). A novel CX3CR1 inhibitor AZD8797 facilitates early recovery of rat acute spinal cord injury by inhibiting inflammation and apoptosis. Int. J. Mol. Med..

[89] Zhang S.Z., Wang Q.Q., Yang Q.Q., Gu H.Y., Yin Y.Q., Li Y.D., Hou J.C., Chen R., Sun Q.Q., Sun Y.F. (2019). NG2 glia regulate brain innate immunity via TGF-β2/TGFBR2 axis. BMC Med..

[90] Honjoh K., Nakajima H., Hirai T., Watanabe S., Matsumine A. (2019). Relationship of Inflammatory Cytokines From M1-Type Microglia/Macrophages at the Injured Site and Lumbar Enlargement With Neuropathic Pain After Spinal Cord Injury in the CCL21 Knockout (plt) Mouse. Front. Cell. Neurosci..

[91] Li B., Liu J., Gu G., Han X., Zhang Q., Zhang W. (2020). Impact of neural stem cell-derived extracellular vesicles on mitochondrial dysfunction, sirtuin 1 level, and synaptic deficits in Alzheimer’s disease. J. Neurochem..

[92] Yamamoto M., Kim M., Imai H., Itakura Y., Ohtsuki G. (2019). Microglia-Triggered Plasticity of Intrinsic Excitability Modulates Psychomotor Behaviors in Acute Cerebellar Inflammation. Cell Rep..

[93] Rodriguez A.R., de Sevilla Müller L.P., Brecha N.C. (2014). The RNA binding protein RBPMS is a selective marker of ganglion cells in the mammalian retina. J. Comp. Neurol..

[94] Niesman I.R., Schilling J.M., Shapiro L.A., Kellerhals S.E., Bonds J.A., Kleschevnikov A.M., Cui W., Voong A., Krajewski S., Ali S.S. (2014). Traumatic brain injury enhances neuroinflammation and lesion volume in caveolin deficient mice. J. Neuroinflamm..

[95] Bocquet A., Berges R., Frank R., Robert P., Peterson A.C., Eyer J. (2009). Neurofilaments bind tubulin and modulate its polymerization. J. Neurosci..

